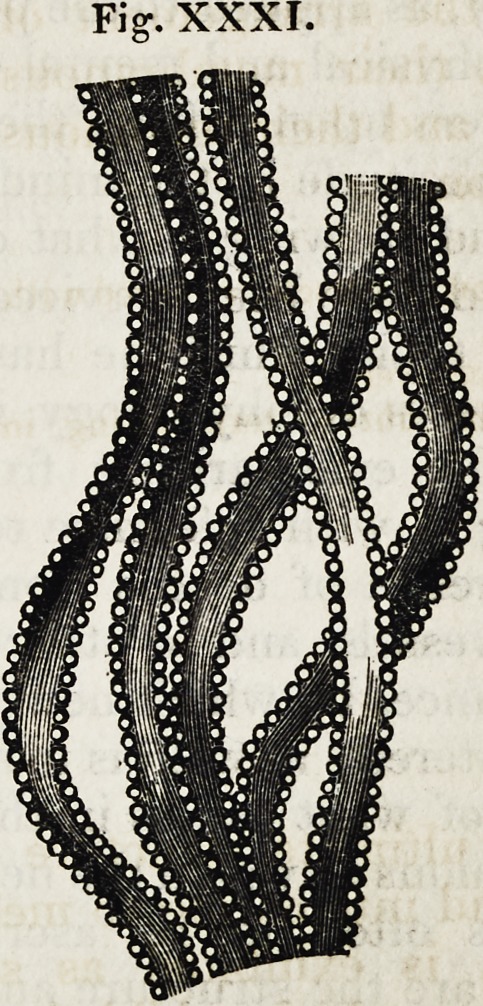# The Philosophy of Health; or, an Exposition of the Physical and Mental Constitution of Man, with a View to the Promotion of Human Longevity and Happiness

**Published:** 1836-04

**Authors:** 


### BRITISH AND FOREIGN
### MEDICAL REVIEW,

FOR APRIL, 1836.

### PART FIRST.

### Analytical and Critical Reviews.

### Art. I.

*The Philosophy of Health; or, an Exposition of the Physical and Mental Constitution of Man, with a View to the Promotion of Human Longevity and Happiness.* By Southwood Smith, m.d., Physician to the London Fever Hospital, to the Eastern Dispensary, to the Jew's Hospital. Vol. I.—*London*, 1835. 8vo. pp. 408.

*The Principles of Physiology applied to the Preservation of Health and to the Improvement of Physical and Mental Education.* By Andrew Combe, m.d., Fellow of the Royal College of Physicians of Edinburgh. *Third Edition, revised and enlarged.—Edinburgh*, 1835. 8vo. pp. 404.

*On the Influence of Atmosphere and Locality; Change of Air and Climate; Seasons; Food: Clothing; Bathing; Exercise; Sleep; Corporeal and Intellectual Pursuits, &c. &c. &c. on Human Health; constituting Elements of Hygiène.* By Robley Dunglison, m.d., Professor of Materia Medica, Therapeutics, Hygiène, and Medical Jurisprudence, in the University of Maryland, &c.—*Philadelphia*, 1835. 8vo. pp. 514.

*Lectures on the Ordinary Agents of Life, as applicable to Therapeutics and Hygiène; or, the Uses of the Atmosphere, Habitations, Baths, Clothing, Climate, Exercise, Food, Drinks, &c. in the Treatment and Prevention of Disease.* By Alexander Kilgour, m.d., Member of the Royal College of Surgeons, London.—*Edinburgh*, 1834. 8vo. pp. 359.

*Lectures on the Means of Promoting and Preserving Health, delivered at the Mechanic's Institute, Spitalfields.* By T. Hodgkin, m d.—*London*, 1835. 8vo. pp. 449.

The therapeutical part of medicine is so imperfect and unsatisfactory, that we perceive with no small satisfaction the direction taken by the authors of these works, whose character places their motives for composing books adapted to general readers far above vulgar suspicion. There is so much, also, in the subject of hygiène, and so much in these excellent publications, deserving a more particular and more habitual attention on the part of medical men, that we feel assured we shall be doing a useful duty to not a few of our readers, by introducing to their observation some of the topics embraced by these well-informed writers. The great ambition of all young practitioners is, we well know, to attain that kind of confidence which will not only ensure them public consideration, but confer upon them the more flattering distinction of being consulted concerning the means of preserving health in families in which there are numerous children; and yet we know how often the young practitioner, who feels not the shadow of a doubt concerning the prompt administration of calomel and colocynth, and senna and rhubarb, is bereft of all his ready wisdom, when the question propounded to him by an anxious parent is, not how to cure an indulged child of a surfeit, but how to prevent, in a succession of children, the supervention of some of the various forms of scrofula. Who is there, long engaged in practice, whose conscience is completely void of having gone on prescribing medicine in certain families for years, at least at frequent intervals, which families were in reality all that time suffering from some local influence, or some rooted error in physical education? Who is there, we may add, who has not on some occasions found it difficult to feel quite as hearty a pleasure as he ought on finding that maladies, which had for months resisted all medicaments, have been expelled by change of air, of food, of exercise, or of occupation,—in short, by *hygiène;* to which he had been, he could not but acknowledge, to himself at least, unaccountably inattentive.

If, indeed, medicine could remedy the many ills against which it is the object of hygienic authors to warn and guard mankind, we might be jealous of so much wisdom being dispersed among unprofessional and common people, and wish to reserve to the medical profession the ancient honours of cure. But, as not a few of the physical evils which men may be taught to avert are of a nature not to admit perfect relief, when once incurred, it is no less worthy of the physician than of the philanthropist to aid that part of mankind whom ignorance makes helpless, by the diffusion of the most salutary kind of knowledge.

If the application of hygiène were even limited to averting the malady which, under the denomination of consumption, carries away in each year so great a number of early victims, (a subject to which our readers' attention was called in the preceding Number, in our review of Dr. James Clark's excellent Treatise,) it would be worthy of the best consideration that could be given to it. But the protection to be expected from hygienic cautions is much more extended, and the benefits they promise may be diffused over communities, and partaken of in every family and by every individual. All classes have, however, up to this period, been ill informed of the particulars on which such benefits depend; and it is a just matter of congratulation, as regards the public, that in the works before us is to be found instruction adapted to each class.

The difficulty of conveying such instruction, so that its reception may be unalloyed by mischief, arises chiefly from the unprepared state of the general reader, who eagerly snatches at practical deductions, with little regard to the reasoning by which they are established. No kind of reading is more universally fascinating than that of medical books; and many diligent persons are learned in symptoms and prescriptions, or the popular practice of physic, who are wholly unacquainted with the structure and functions of the human body; that structure which they boldly attempt to repair, and those functions which they as rashly presume to regulate.

To such persons, comprehending almost all general readers, Dr. Southwood Smith's book presents a variety of most desirable information, expressed in clear and correct language, and well calculated to prepare them for understanding the principles by attending to which health is to be preserved, or, if lost, regained. With the assistance of numerous plates, he has given to the reader an intelligible view of animal structure, professedly preparatory to a second volume, which is to relate exclusively to health and disease.

No person of education can peruse the volume already published without gratification; and we think it will go far to make anatomy and physiology, what they surely ought to be, popular studies. The volume consists of seven chapters; the first four are devoted to certain general observations introductory to the whole subject, and containing considerations so important and interesting that we shall speak of them more fully. The fifth chapter, which fills up about half of the book, is full of details relating to the general and descriptive anatomy of the human body, which the reader will find given in clear and attractive language, illustrated by numerous and highly instructive plates. The sixth chapter relates to the characters of the blood; and in the seventh, the circulation is described.

As our notice is at present drawn to this interesting work on account of its connexion with the means of preserving the health of individuals and communities, we are precluded from giving any of the many examples that might be selected of the author's powers of description, and can but speak generally of merits on which we could with pleasure dwell in detail. No author within our range of reading, certainly no popular author, has so strongly placed before his readers the great ends to be attained by the preservation of health, or those general views which give importance to the subject of hygiène.

In a very lucid and eloquent chapter, (the first,) Dr. Southwood Smith lays down the characters which distinguish living beings from inorganic bodies, and animals from plants; the latter subject being admirably illustrated by such simple examples as the most unprepared reader can understand; and he briefly and lucidly defines the principles of the greater complexity of animal structure, both in relation to plants and to superiority of function or greater energy of existence. A child, by reading this chapter, might be made acquainted with some of the chief general truths of natural history; and yet the elegance and force of the language would recommend it to the most cultivated reader. We have seldom read any thing surpassing in precision, both as to matter and language, the chapter which follows, in which are explained the diversities in the modes of organic and animal life: and the real difficulty of quoting here consists in this, that we should not know where to leave off. Every reader who advances thus far into the book will, we think, be of our opinion, that, by extent of knowledge, readiness of illustration, and force and propriety of language, Dr. Southwood Smith is eminently qualified for the task of diffusing philosophical truths; and in the present state, and with the present prospects of society, a higher task can hardly be conceived. Familiar as many of the circumstances mentioned are to all medical readers, they are presented even to them in a form which gives them almost the air of novelty, as in the following passage, in which, after noticing some of the general anatomical distinctions between the organs of organic and of animal life, the author thus expresses himself:

"In general, the apparatus of the organic life is placed in the interior of the body, while that of the animal life is placed on the external surface. The organic organs are the instruments by which life is maintained. There is no action of any one of them that can be suspended, even for a short space of time, without the inevitable extinction of life. But the animal organs are not so much instruments of life as means by which a certain relation is established between the living being and external objects: and this difference in their office is the reason of the difference in their position. Existence depending on the action of the organic organs, they are placed in the interior of the body; they are fixed firmly in their situation, in order that they may not be disturbed by the movements of locomotion; they are enveloped in membranes, covered by muscles, placed under the shelter of bones, and every possible care is taken to secure them from accident, and to shield them from violence. Existence not being immediately dependent on the action of the organs of the animal life, they do not need to be protected from the contact of external objects with extraordinary care; but it is necessary to the performance of their functions that they should be placed at the exterior of the body. And there they are placed, and so placed as to afford an effectual defence to the organic organs. Thus the groundwork of the animal is made the bulwark of the organic life. The muscles, the immediate agents by which voluntary motion is effected, and the bones, the fixed points and the levers by which that motion acquires the nicest precision, and the most prodigious rapidity and power, are so disposed that, while the latter accomplish, in the most perfect manner, their primary and essential office in relation to the muscles, they serve a secondary but scarcely less important office in relation to the internal viscera. As we advance in our subject, we shall see that a beautiful illustration of this is afforded in the structure and action of the trunk; that the trunk is moveable; that it is composed of powerful muscles, and of firm and compact bones; and that, while its movements are effected by the action of the muscles which are attached to the bones, these bones enclose a cavity, in which are placed the lungs, the heart, the great trunks of the venous system, the great trunks of the arterial system, and the main trunk of the thoracic duct, the vessel by which the digested aliment is carried into the blood. (Chap. 5.) Thus, by these strong and firm bones, together with the thick and powerful muscles that rest upon them, is formed a secure shelter for a main portion of the apparatus of the organic functions of respiration, circulation, and digestion. The bones and muscles of the thorax, themselves performing an important part in the function of respiration, afford to the lungs, the chief organ of this function, composed of tender and delicate tissues, easily injured, and the slightest injury perilling life, a free and secure place to act in. The fragile part of the apparatus is defended by the osseous portion of it, the play of the latter being equally essential to the function as that of the former. In like manner the tender and delicate substance of the brain and spinal cord, the central seat of the animal life, with which all the senses are in intimate communion, is protected by bones and muscles, which perform important voluntary movements; while the organs of sense, which put us in connexion with the external world, which render us susceptible of pleasure, and which give us notice of the approach of objects capable of exciting pain, are placed where external bodies may be brought most conveniently and completely into contact with them; and where alone they can be efficient as the sentinels of the system. For this reason, with the exception of the sense of touch, which, though placed especially at the extremities of the fingers, is also diffused over the whole external surface of the frame, all the senses have their several seats in the head, the most elevated part of the body, of an ovoid figure, capable of moving independently of the rest of the fabric, and which, being supported on a pivot, is enabled to describe at least two-thirds of a circle.

"Such is the difference in the structure and position of the apparatus of the two lives, but the difference in their action is still more striking." (P. 52.)

Very few of the particulars here presented to the medical reader's attention are such as have not been attended to by him before; but they are strikingly expressed, and so as to leave no general reader unsatisfied with the argument conveyed by them.

Dr. Smith then proceeds to describe the difference in the action of these two systems of organs; the unconscious and unfailing movements of the organic apparatus; and the conscious exercise of the animal functions, with their need of relaxation or of rest; the close relation and dependence of all the functions of organic life; the comparative independence of the animal functions of one another, or at least their capability of separate lesion, separate action, separate repose; the existence of the organic before the animal life, and their primary perfection, contrasting this with the slow attainment of a full exercise of the functions of animal life, as of the actions of the voluntary muscles, the sensations, and the intellectual operations; and, lastly, the circumstances of disease or age in which the animal life is extinguished, and the organic life survives.

In the third chapter of the work we have a condensed view of the apparent objects of all the apparatus before spoken of and subsequently described; in other words, the ultimate objects of organization and life; and these are stated to be pleasure, enjoyment, happiness. We believe we shall not be incorrect in saying, that this chapter contains a kind of summary of what has been much more talked of in late years than well considered, except by a very small but most respectable sect, the followers of the venerable and philanthropic Bentham;—we mean *the greatest happiness principle;* to which Dr. Smith brings the not inapt illustrations derivable from physiology. Structure, he says, is created for the performance of functions; functions are organic and animal; the organic functions are intended for maintaining the condition essential to the performance of the animal functions. Animal life comprises two functions, sensation and voluntary motion, and voluntary motion is the mere servant of sensation.

"Is sensation, then," he continues, "the ultimate object of organization? Simple sensation cannot be an ultimate object, because it is invariably attended with an ultimate result; for sensation is either pleasurable or painful. Every sensation terminates in a pleasure or a pain. Pleasure or pain, the last event in the series, must then be the final end.

"Is the production of pain the ultimate object of organization? That cannot be; for the production of pain is the indirect, not the direct,—the extraordinary, not the ordinary, result of the actions of life. It follows that pleasure must be the ultimate object; for there is no other of which it is possible to conceive. The end of organic existence is animal existence; the end of animal existence is sentient existence; the end of sentient existence is pleasurable existence; the end of life, therefore, is enjoyment. Life commences with the organic processes; to the organic are superadded the animal; the animal processes terminate in sensation; sensation ends in enjoyment; it follows, that enjoyment is the final end. For this every organ is constructed; to this every action of every organ is subservient; in this every action ultimately terminates.

"And, without a single exception in the entire range of the sentient creation, the higher the organized structure the greater the enjoyment, mediately or immediately, to which it is subservient. From its most simple to its most complex state, every successive addition to structure, by which function is rendered more elevated and perfect, proportionally increases the exquisiteness of the pleasure to which the function ministers, and in which it terminates." (P. 74.)

These views are followed by various appropriate observations. The pleasurable consciousness of the proper performance of the organic functions, or what has been called the sense of well-being, although in states of health the organs of those functions do not possess common sensation, which would be useless to us, is not only dwelt on in support of such views, but shewn to be dependent on the anatomy of the nervous system, and illustrated by a figure representing the origins and connexions of some of the spinal nerves and the great sympathetic. In imparting or in limiting sensation, comfort, or pleasure, happiness is shewn to be equally the object. The pleasures of sense are of course pointed to as evincing the same design; and the higher pleasures of the intellect are dwelt upon with equal eloquence and truth. What are termed the sympathetic pleasures are placed in strong contrast to the selfish:—

"As the organic life produces and sustains the animal, so the sympathetic principle is produced and sustained by the selfish. As the organic life is conservative of the entire organization of the body, so the selfish principle is conservative of the entire being. As the animal life is superadded to the organic, extending, exalting, and perfecting it, so the sympathetic principle is superadded to the selfish, equally extending, exalting, and perfecting it. The animal life is nobler than the organic, whence the organic is subservient to the animal; but there is not only no opposition, hostility, or antagonism between them, but the strictest possible connexion, dependence, and subservience. The sympathetic principle is nobler than the selfish, whence the selfish is subservient to the sympathetic; but there is not only no opposition, hostility, or antagonism between them, but the strictest possible connexion, dependence, and subservience. Whatever is conducive to the perfection of the organic, is equally conducive to the perfection of the animal life; and whatever is conducive to the attainment of the true end of the selfish is equally conducive to the attainment of the true end of the sympathetic principle." (P. 91.)

Arguing still for harmony between these two principles, the selfish and the sympathetic, Dr. Smith goes on to state that it is the office of the moral faculty to discover whatever is productive in sensation, emotion, affection, or action, of real instead of delusive pleasure, of pure instead of mixed pleasure, and of lasting instead of temporary pleasure; and that the operation of this faculty, when correct and complete, enables the human being to enjoy the maximum of felicity. In this sense, he observes, virtue is happiness. Dwelling on this interesting subject, he expresses an opinion, doubtless containing much truth, and of much importance, when reflected upon in all its consequences,—that *there is a close connexion between happiness and longevity.*

"Enjoyment is not only the end of life, but it is the only condition of life which is compatible with a protracted term of existence. The happier a human being is, the longer he lives; the more he suffers, the sooner he dies; to add to enjoyment, is to lengthen life; to inflict pain, is to shorten the duration of existence. As there is a point of wretchedness beyond which life is not desirable, so there is a point beyond which it is not maintainable. The man who has reached an advanced age cannot have been, upon the whole, an unhappy being; for the infirmity and suffering which embitter life cut it short. Every document by which the rate of mortality among large numbers of human beings can be correctly ascertained, contains in it irresistible evidence of this truth. In every country, the average duration of life, whether for the whole people or for particular classes, is invariably in the direct ratio of their means of felicity; while, on the other hand, the number of years which large portions of the population survive beyond the adult age may be taken as a certain test of the happiness of the community. How clear must have been the perception of this in the mind of the Jewish legislator when he made the promise, THAT THY DAYS MAY BE LONG IN THE LAND WHICH THE LORD THY GOD HATH GIVEN THEE,—the sanction of every religious observance, and the motive to every moral duty." (P. 101.)

Willing as we are to lend our belief to the principal observation which the above passage conveys, that longevity is closely connected with the enjoyment of mental and bodily comfort, we cannot but remark that there appears to be, both in this and some preceding passages, too obvious a putting aside of any motives to purity of conduct arising out of a contemplation of the source of all the structures and functions there discoursed of, "the fountain of all goodness," the "*Universal* Good," which, notwithstanding a thousand different views of his essential nature, to us hidden and incomprehensible, or dimly seen in these his works, may be the object of universal adoration. The tendency, too, in human beings, to uplift their views towards creatures supposed to realize the perfection at which hitherto mortals have vainly aimed, and the general belief that has grown up in all corners of the earth, that all which we here call life is prefatory to some higher existence, are attributes of our nature which the physiologist cannot overlook. The same may be said of our power of improving the mental faculties and the moral qualities, which seems to connect us with a higher rank of existence, to which we may suppose that we are proceeding. If we exclude all the sense of pleasure arising from a belief that in selecting true from false happiness, so as to secure virtue, we act conformably to the will of the Providence which sustains the great world, is there not some danger lest the selection may be too frequently governed by the selfish principle, clothing itself in the garb of the sympathetic? The tenor of these remarks must shew how remotely we wish to steer from purely religious controversy; and we shall not even dwell longer on what may be thought approaching to metaphysical discussion; but the omission to which we have alluded will no doubt limit the number of Dr. Smith's readers. We know that a critic must often be ignorant of the precise motives for the plan followed by his author, and are willing to allow that Dr. Smith may have had good reasons for overleaping considerations which so consecutive a reasoner could not but have found at one time straight before him. The omission is the more remarkable in an author who has already achieved no inconsiderable reputation by a work upon the Divine Government, which has been pronounced by eminent critics to be equally eloquent and convincing.

Those to whom Dr. Smith's opinions, as expressed in the work before us, would be unexceptionable, must have convinced themselves, like certain sects of ancient philosophers, that man is fully equal to the attainment of perfect happiness in this world, and that what we call the trials of human life are *all* avoidable, and in no degree necessary to moral improvement. To declare hostility to such opinions would expose us to a contest with able gladiators, in an arena which we have no inclination to enter; whilst, to avow how far we go along with them in their prospective views of man's physical and moral amendment would perhaps equally expose us to the charge of optimism.

The conclusion of the chapter from which the above passages are taken is, at all events, too characteristic of the philosophic sect which we set out by mentioning to be omitted. To many mere medical readers, we believe it will present some new matter. To us it seems correct and beautiful; and it furnishes an answer, founded on the past progress of man's knowledge of physical and of moral science, to those who are always too ready to suppose he has already nearly reached the bounds beyond which, in his mysterious progress, he will not be permitted to pass.

"Deeply, then, are laid the fountains of happiness in the constitution of human nature. They spring from the depths of man's physical organization; and, from the wider range of his mental constitution, they flow in streams magnificent and glorious. It is conceivable that, from the first to the last moment of his existence, every human being might drink of them to the full extent of his capacity. Why does he not? The answer will be found in that to the following question: What must happen before this be possible? The attainment of clear and just conceptions on subjects, in relation to which the knowledge hitherto acquired by the most enlightened men is imperfect. Physical nature, every department of it, at least, which is capable of influencing human existence and human sensation; human nature, both the physical and the mental part of it; institutions so adapted to that nature as to be capable of securing to every individual, and to the whole community, the maximum of happiness with the minimum of suffering—this must be known. But knowledge of this kind is of slow growth. To expect the possession of it on the part of any man in such a stage of civilization as the present, is to suppose a phenomenon to which there is nothing analogous in the history of the human mind. The human mind is equally incapable of making a violent discovery in any department of knowledge, and of taking a violent bound in any path of improvement. What we call discoveries and improvements are clear, decided, but for the most part gentle, steps in advancement for the actual and immediately preceding state of knowledge. The human mind unravels the great chain of knowledge link by link: when it is no longer able to trace the connecting link, it is at a stand; the discoverer, in common with his contemporaries, seeing the last ascertained link, and by that led on by analogies which are not perceived by, or which do not impress, others, at length descries the next in succession: this brings into view new analogies, and so prepares the way for the discernment of another link; this again elicits other analogies, which lead to the detection of other links, and so the chain is lengthened. And no link, once made out, is ever lost.

"Chemists tell us that the adjustment of the component elements of water is such, that, although they readily admit of separation, and are subservient to their most important uses in the economy of nature by this very facility of decomposition, yet that their tendency to recombination is equal; so that the quantity of water actually existing at this present moment in the globe is just the same as on the first day of creation: neither the operations of nature, nor the purposes to which it has been applied by man, having used up, in the sense of destroying, a single particle of it. Alike indestructible are the separate truths that make up the great mass of human knowledge. In their ready divisibility, and their manifold applications, some of them may sometimes seem to be lost; but, if they disappear, it is only to enter into new combinations, many of which themselves become new truths, and so ultimately extend the boundaries of knowledge. Whatever may have been the case in time past, when the loss of an important truth, satisfactorily and practically established, may be supposed possible, such an event is inconceivable now, when the art of printing at once multiplies a thousand records of it, and, with astonishing rapidity, makes it part and parcel of hundreds of thousands of minds. A thought more full of encouragement to those who labour for the improvement of their fellow-beings, there cannot be. No onward step is lost; no onward step is final; every such step facilitates and secures another. The savage state,—that state in which gross selfishness seeks its object simply and directly by violence,—is past. The semi-savage or barbarous state, in which the grossness of the selfishness is somewhat abated, and the violence by which it seeks its object in some degree mitigated, by the higher faculties and the gentler affections of our nature, but in which war still predominates, is also past. To this has succeeded the state in which we are at present, the so-called civilized state,—a state in which the selfish principle still predominates, in which the justifiableness of seeking the accomplishment of selfish purposes by means of violence,—that of war among the rest,—is still recognized, but in which violence is not the ordinary instrument employed by selfishness, its ends being commonly accomplished by the more silent, steady, and permanent operation of institutions. This state, like the preceding, will pass away. How soon, in what precise mode, by what immediate agency, none can tell. But we are already in possession of the principle which will destroy the present, and introduce a better social condition, namely, the principle at the basis of the social union,—THE MAXIMUM OF THE AGGREGATE OF HAPPINESS; THE MAXIMUM OF THE AGGREGATE OF HAPPINESS SOUGHT BY THE PROMOTION OF THE MAXIMUM OF INDIVIDUAL HAPPINESS!" (P. 102.)

Not content with the mere enunciation of the opinions above mentioned, respecting the connexion between happiness and longevity, Dr. Southwood Smith has devoted the fourth chapter of his work to certain statistical results, which go far to prove their correctness. He refers to the ordinary observation of every one, in proof of the common influences of good and bad fortune on the appearance of individuals; and he quotes from M. Villermé a statement that the ordinary mortality in the prisons of France is one in twenty-three, although a large majority of the prisoners are between twenty-five and forty-five years of age; and that the mortality of the indigent class throughout the country is double that of the wealthy.

The following considerations are ingenious, and calculated to interest every reader.

"An advanced term of life and decrepitude are commonly conceived to be synonimous: the extension of life is vulgarly supposed to be the protraction of the period of infirmity and suffering; that period which is characterized by a progressive diminution of the power of sensation, and a consequent and proportionate loss of the power of enjoyment; the "sans teeth, sans eyes, sans taste, sans every thing." But this is so far from being true, that it is not within the compass of human power to protract, in any sensible degree, the period of old age, properly so called,—that is, the stage of decrepitude. In this stage of existence, the physical changes that successively take place clog, day by day, the vital machinery, until it can no longer play. In a space of time, fixed within narrow limits, the flame of life must then inevitably expire; for the processes that feed it fail. But though, when fully come, the term of old age cannot be extended, the coming of the term may be postponed. To the preceding stage, an indefinite number of years may be added; and this is a fact of the deepest interest to human nature.

"The division of human life into periods or epochs is not an arbitrary distinction, but is founded on constitutional differences in the system, dependent on different physiological conditions. The periods of infancy, childhood, boyhood, adolescence, manhood, and old age, are distinguished from each other by external characters, which are but the outward signs of internal states. In physiological condition, the infant differs from the child, the child from the boy, the boy from the man, and the adult from the old man, as much in physical strength as in mental power. There is an appointed order in which these several states succeed each other; there is a fixed time at which one passes into another. That order cannot be inverted; no considerable anticipation or postponement of that fixed time can be effected. In all places, and under all circumstances, at a given time, though not precisely at the same time in all climates and under all modes of life, infancy passes into childhood, childhood into boyhood, boyhood into adolescence, and adolescence into manhood. In the space of two years from its birth, every infant has ceased to be an infant, and has become a child; in the space of six years from this period, every male child will have become a boy; add eight years to this term, and every boy will have become a young man; in eight years more, every young man will have become an adult man; and, in the subsequent ten years, every adult man will have acquired his highest state of physical perfection. But at what period will this state of physical perfection decline? What is the maximum time during which it can retain its full vigour? Is that maximum fixed? Is there a certain number of years in which, by an inevitable law, every adult man necessarily becomes an old man? Is precisely the same number of years appointed for this transition to every human being? Can no care add to that number? Can no imprudence take from it? Does the physiological condition or the constitutional age of any two individuals ever advance to precisely the same point in precisely the same number of years? Physically and mentally are not some persons older at fifty than others are at seventy? And do not instances occasionally occur in which an old man, who reaches even his hundredth year, retain as great a degree of juvenility as the majority of those who attain to eighty?

"If this be so, what follows? One of the most interesting consequences that can be presented to the human mind. The duration of the periods of infancy, childhood, boyhood, and adolescence, is fixed by a certain number of years. Nothing can stay, nothing retard, the succession of each. Alike incapable of any material protraction is the period of old age. It follows that every year by which the term of human existence is extended is really added to the period of mature age; the period when the organs of the body have attained their full growth, and put forth their full strength; when the physical organization has acquired its utmost perfection; when the senses, the feelings, the emotions, the passions, the affections, are in the highest degree acute, intense, and varied; when the intellectual faculties, completely unfolded and developed, carry on their operations with the greatest vigour, soundness, and continuity: in a word, when the individual is capable of receiving and of communicating the largest amount of the highest kind of enjoyment.

"A consideration more full of encouragement, more animating, there cannot be. The extension of human life, in whatever mode and degree it may be possible to extend it, is the protraction of that portion of it, and only of that portion of it, in which the human being is capable of RECEIVING AND OF COMMUNICATING THE LARGEST MEASURE OF THE NOBLEST KIND OF ENJOYMENT." (P. 111.)

We have quoted largely from this portion of Dr. Smith's work, because it relates to subjects a little out of the ordinary path pursued by physiologists, and which are stated in a manner likely to procure them at least a dispassionate consideration. For an account of the support given to these animating views of protracted life, afforded by an observation of the actual numbers that die at different ages, we must refer the reader to the chapter from which the preceding passages are taken, and which is full of interest for all to whom the value of life, the reduction of mortality, the increased happiness of the people, and the causes that are producing, and that may be expected to produce further, most desirable results, are proper objects of study. The latter branch of enquiry, or that of the *causes*, is postponed by Dr. Smith to a subsequent volume, and will, we doubt not, be investigated with the same freedom and ability by which the pages of his first volume are distinguished.

Having disposed of these preliminary subjects, Dr. Smith enters, in his fifth chapter, on a description of the structure and functions of the human body, which are explained with a fulness that obviates any accusation of the description being superficial, and yet with a brevity which must prevent any intelligent reader from charging the author with being tedious. We know of no work in which so much general and special anatomy is conveyed in so small a compass, and so intelligibly, and so agreeably. It would be a pleasant task to quote some specimens of the instructive manner in which anatomical and physiological truths are set before the general reader; but, as the descriptions of course comprehend subjects familiar to the medical reader, it might be deemed a work of supererogation so to do. An idea of the copiousness of the illustrations may be afforded by our stating that twenty-two figures are introduced to assist the portion of the text describing the anatomy and movements of the arm and hand. Seeing how powerfully these figures aid the description, we cannot but wonder that the professional student so long remained poorer in helps of this sort than is now the general reader, and look back with surprise upon the unenlivened labours of the closet, by which the industrious learner of anatomy used of old to endeavour to advance faster than the mere lessons of the lecture-room and scanty dissections admitted. One difficulty, however, will be felt by the general student in the perusal of Dr. Smith's work, from the author's exclusive employment of the scientific nomenclature used by the anatomists. This might be obviated by a glossary, explaining, for instance, that scapula means shoulder-blade; clavicle, collar-bone; olecranon, the end of the bone which forms the point of the elbow; the humerus, the bone extending from the shoulder to the elbow, &c. &c.

Fig. XXIII.

To our medical readers, few of the illustrative figures would present, perhaps, any novelty; but it may serve to explain Dr. Smith's manner of aiding his descriptions, if we transfer a few of them to our pages. The fact of the softening of a bone, when the osseous portion is destroyed, although the membranous portion may yet retain the original form of the bone, is impressed upon the reader by a representation of the bone in its membranous condition, in which state it is capable of being twisted into a knot.

Another figure represents the reverse state of the bone; when its membranous portion has been destroyed by fire, and the earthy part of the bone remains unchanged.

Fig. XXIV.

The structure of a muscle, concerning which it is often difficult to give a clear notion to an unprofessional auditor, is readily explained by a figure showing the arrangement of its fibres, their membranous envelope, and their tendinous termination.

Fig. XXVI.

*a*. A portion of muscle, covered with membrane.
*b*. Ditto, uncovered.
*c*. Muscular fibres, terminating in tendon.

Fig. XXVIII.

And the ultimate structure of muscles, alike curious and interesting to medical and general enquirers, is exhibited as seen through the microscopes, which exhibit it as consisting of a series of rounded particles, or globules, like a string of pearls, each globule being commonly stated to be about the two-thousandth part of an inch in diameter.

But, to prevent an erroneous opinion, which, apparently dependent on ocular proof, might be with difficulty corrected, Dr. Smith informs the reader, that, under microscopes still further improved, these globules disappear, and the muscular tissue appears "as a peculiar pulpy substance, arranged into threads of extreme minuteness, placed close and parallel to each other, intersected by a great number of delicate lines passing transversely across the muscular threads;" and this appearance, as seen under Mr. Lister's microscope, when the object is magnified five hundred diameters, is shewn by

Fig. XXIX.

The two following illustrations are introduced, among others, to convey an idea of the general appearance and the ultimate tissue of nerves.

Fig. XXX.

Fig. XXXI.

We well remember a time when even students of medicine would have been glad to obtain such information, and in such a manner.

In the author's dedication of this first volume to Lord Brougham, whose services in the cause of liberal education are so well known as to make any eulogium from us wholly unnecessary, he points out the lamentable fact, that in no school or college in England are included, in the curriculum of study, an explanation of the structure and functions of the human body, or of the phænomena of the human mind, and of the laws that govern the formation and direction of the intellectual and moral powers. He justly enforces the importance of such parts of study to lawyers, to magistrates, and to legislators, mentioning some of the results of the want of this kind of knowledge in such functionaries, which might, indeed, have been greatly increased in number. It will be well for societies when such studies attract more general attention; and we think Dr. Southwood Smith's work can hardly fail to contribute to this desirable effect.

Dr. Combe's treatise has already been widely circulated, and has passed to a third edition within a year from its first appearance; and it has been republished and stereotyped in America. After setting forth, in a preface, the true importance of the subject of physiology, as applied to the preservation of health and the improvement of physical and mental education, and defended himself and other writers upon it from the possible charge of creating an unnecessary solicitude in the minds of general readers on the subject of health; and shewing in what circumstances some knowledge of this kind would have been serviceable to the public; Dr. Combe introduces the topics which he has selected for illustration, by remarks on the objects of physiology in general; and speaks more particularly of the evils arising from the general ignorance prevalent respecting it, with reference to the Factories' Regulation Bill, and the occurrence of deaths from want of a proper supply of air on board of vessels, and in other situations; and to the existence of a law in France, by which newly-born infants are taken to the mayor to be registered; as well as to a number of examples familiar to most observers of what passes in the rooms of the sick, and to the erroneous opinions to which the neglect of health and the origin of many diseases is often to be ascribed. The topics then particularly treated of are the structure and functions of the skin, the nature of the muscular system, the structure and uses of the bones, and of the lungs; the nature of the nervous system, and the faculties of the mind. To the descriptive part of each of these subjects are appended remarks on the rules by the observance of which each of them may be kept in health, and may conduce to the general health of the body. The rules are shewn to be sensible and judicious by a continual reference to the explanatory portions of the work; and thus the reader is led to wholesome customs, by being taught the reason of their being wholesome. It appears to have been Dr. Combe's first intention, in the selection of his topics, to comprehend such functions as were not only most influential in their operation, but least familiarly known: he states, however, in a note in the third edition, that he is preparing to comply with suggestions which have been frequently made to him, to give a similar account of the functions of digestion, nutrition, the circulation, &c.; and this we are glad to learn, as the popularity of his first volume will ensure the introduction of his second into innumerable families, wherein the valuable information and advice which characterizes his writings will be of material service.

As, next to what concern the function of digestion, no errors are more prevalent than those which might be corrected by a knowledge of the benefit of attending to the condition of the surface, we regard Dr. Combe's first and second chapters, which relate to this part of physiology, as likely to be particularly serviceable to his readers. The first chapter is wholly descriptive of the cuticle, the mucous coat, and the true skin, and contains little which our readers can be supposed to be ignorant of or to have forgotten, but the description is interspersed with remarks which not only relieve it, but usefully exercise the reader's mind. Speaking, for instance, of so simple and common a circumstance as the thickening of the cuticle, it is well observed that

"The greater thickness of the cuticle in such situations is manifestly the intentional work of the Creator; for it is perceptible even at birth, before use can have exercised any influence. Indeed, were the tender skin not so protected, every violent contraction of the hand upon a rough and hard surface, and every step made on uneven ground, would cause pain, and disable us for exertion.

"By another beneficent provision, calculated to afford increased protection, according to the necessities of the individual, it happens that, when a part is much used, the cuticle covering it becomes thicker and thicker within certain limits, till, in extreme cases, it becomes as thick, hard, and resisting, as horn. It is this thickening of the epidermis on the lady's finger that alone enables her to wield with impunity that important instrument, the needle; and it is the same thickening that fits the blacksmith and the mason, the stonebreaker and the boatman, to ply their trades, without that painful blistering which the young apprentice or unaccustomed labourer so regularly undergoes, and which must have continued to recur for ever, had the cuticle been organized with blood-vessels and nerves, or not subjected to this law of becoming thicker wherever increased protection is required.

"Another modification of the cuticle to suit a modification of circumstances, is that observed in the nails. These belong to the scarf-skin, and separate with it; and, like it, they have neither blood-vessels or nerves, and may be cut or bruised without pain. When the hand or foot is macerated in water, the nails and the cuticle show their identity of organization, by separating together from the dermis or true skin below. The nails, like the cuticle, serve chiefly to protect the subjacent parts from injury; and, accordingly, in those lower animals whose manner of life subjects their feet to continual pressure, and requires no nice exercise of touch, nature has provided horny and resisting hoofs for their protection, instead of a merely thickened epidermis.

"To produce thickening of the cuticle, exercise must be gradual, and not too severe. If, for example, a person takes a very long walk, rows a boat, or makes use of a heavy hammer, for a few hours, without having been accustomed to such an effort, there is no time for the cuticle to thicken and defend itself from the unusual friction. The parts below, being inadequately protected, become irritated and inflamed, and throw out a quantity of watery fluid or serum on their surface, which raises up the cuticle in blisters, and, by making it painful to continue the pressure, obliges the person to desist from an exercise, which, if continued, would evidently soon alter the structure of the sentient nervous filaments, and for ever unfit them for their proper uses. So that, even in this result, beneficence and wisdom are prominently displayed." (P. 43.)

Observations of this kind awaken every reader's attention, by shewing that in all which surrounds us, and in every part of the corporeal fabric, there is matter for reflection and enquiry; that there is nothing trivial in the system of which the corporeal fabric forms a part. Thus, also, in speaking of the mucous coat, Dr. Combe glances at the interesting fact that this coat is the seat of the magnificent colouring of the skin of many fishes and other animals, in which it has often almost a metallic splendour: and he at once conveys the impression of the undoubted importance of the true skin and its functions, by describing the average extent of its surface as 2500 square inches, and this extent so crowded with blood-vessels and with nerves as almost to seem a network of them; and in this manner he prepares every reader to consider the extensive influence of it, "1st, as an exhalent of waste matter from the system; 2dly, as a joint regulator of the heat of the body; 3dly, as an agent of absorption; and, 4thly, as the seat of sensation and touch." Each of these properties is well explained; the chief facts connected with each are simply stated, and the inferences are clearly and convincingly attached to them. The office of the skin as an exhalent organ is not only calculated to surprise a reader previously unacquainted with the subject, but he may gather serviceable hints, of daily application, from that which occasions his surprise. The necessity of attending to the state of the surface is seen at once, on the announcement that from the surface alone at least twenty ounces of waste matter are removed from the system in every twenty-four hours, in a state invisible to the eye. The evils which may follow a checked perspiration, or long exposure of the skin to cold, can never be forgotten by those who have learnt this simple fact: the unprofessional reader will no longer be disposed to look upon extensive cutaneous affections, measles, or small-pox, for instance, as things to be lightly thought of, and the serious injury ensuing in internal organs after an extensive burn on the surface becomes explained: and all this knowledge it is most desirable that the public should possess, for it is not of the kind which leads to quackery, but rather to a more just appreciation of the talents of a practitioner, and more reasonable expectations from the employment of remedial means in the hands of the well informed.

In the same way, the statements given by Dr. Combe respecting the part borne by the skin in regulating animal temperature, bring with them salutary suggestions applicable to those exposed to the influence of warm climates, or of the night air in our own; and the account given of its absorbing power is connected with momentous precautions respecting dress, and exposure to concentrated and unwholesome effluvia or miasmata. How many interesting practical points are comprehended, for instance, in the following extract. The professional reader, even, cannot be reminded of them without advantage.

"When the perspiration is brought to the surface of the skin, and confined there either by injudicious clothing or by want of cleanliness, there is much reason to suppose that its residual parts are again absorbed, and act on the system as a poison of greater or less power, according to its quantity and degree of concentration; thereby producing fever, inflammation, and even death itself; for it is established by observation, that concentrated animal effluvia form a very energetic poison. The fatal consequences which have repeatedly followed the use of a close water-proof dress by sportsmen and others, and the heat and uneasy restlessness which speedily ensues where proper ventilation is thus prevented, seem explicable on some such principle.

"It is believed by many that marsh miasmata, and other poisons, are absorbed by the skin; and Bichat considered the fact as established in regard to the effluvia of dissecting-rooms. There are many reasons for concurring in this belief. The plague, for instance, is known to be much more readily communicated by contact than by any other means, and this can happen only through the medium of absorption. Again, it is certain that flannel and warm clothing are extremely useful in preserving those who are unavoidably exposed to the action of malaria and of epidemic influences; and these manifestly act chiefly by protecting the skin. A late writer on the Malaria of Rome strongly advocates this opinion, and expresses his conviction that the ancient Romans suffered less from it, chiefly because they were always enveloped in warm woollen dresses. This opinion, he says, is justified by the observation, that, since the period at which the use of woollen clothing came again into vogue, intermittent fevers have very sensibly diminished in Rome. Even in the warmest weather, the shepherds are now clothed in sheepskins. Brocchi, who experimented extensively on the subject, obtained a notable quantity of putrid matter from the unwholesome air, and came to the conclusion that it penetrated by the pores of the skin, rather than by the lungs. Brocchi ascribes the immunity of the sheep and cattle, which pasture night and day in the Campagna, to the protection afforded them by their wool. These remarks deserve the serious attention of observers, particularly as, according to Patissier, similar means have been found effectual in preserving the health of labourers, digging and excavating drains and canals in marshy grounds, where, previous to the employment of these precautions, the mortality from fever was very considerable.

"It is a general law, that every organ acts with increased energy when excited by its own stimulus; and the application of this law to the different functions of the skin may help to remove some of our difficulties. The skin exhales most in a warm dry atmosphere, because the latter dissolves and carries off the secretion as fast as it is produced; and the same condition is unfavorable to absorption, because nothing is present upon which the absorbents of the skin can act. In a moist atmosphere, on the other hand, the absorbents meet with their appropriate stimulus, and act powerfully; while exhalation is greatly diminished, because the air can no longer carry off the perspiration so freely. Apparently from this extensive absorption, we find the inhabitants of marshy and humid districts remarkable for the predominance of the lymphatic system, as has long been remarked of the Dutch; and, as malaria prevails chiefly in situations and seasons in which the air is loaded with moisture, and is most energetic at periods when absorption is most active, and moisture is at its maximum, the probability of its being received into the system chiefly by cutaneous absorption is greatly increased, and the propriety of endeavouring to protect ourselves from its influence by warm woollen clothing becomes more striking. In the army and navy, accordingly, where practical experience is most followed, the utmost attention is now paid to enforcing the use of flannel and sufficient clothing, as a protection against fever, dysentery, and other diseases, particularly in unhealthy climates. In the prevention of cholera, flannel was decidedly useful.

"From the above exposition of the laws of absorption, and from the facts referred to at page 64, may it not be feasibly inferred, that the efficacy of great heat in preventing contagion from the plague is partly owing to the consequent dryness of the atmosphere no longer presenting the requisite stimulus to the absorbents, but, on the contrary, powerfully exciting the action of the exhalants? Damp directly stimulates the absorbents, and hence may arise its hurtfulness as a vehicle. The system, too, it is well known, is peculiarly susceptible of infection when the stomach has been for some time empty, as before breakfast. May not this be accounted for by the then greater activity of absorption?" (P. 67.)

The chapter which follows the description of the skin, and which relates to its health, and the influence it exercises on the general system, is perhaps one of the most important in the book; neglect of clothing suited to our climate and to the different seasons being one of the most common of faults, and one of the most detrimental to the inhabitants of this island. This is particularly exemplified in the dress of infants and young persons, and often, indeed, in people of every age up to an advanced period of life. As regards little girls and young women, the neglect of such dress as affords sufficient protection from cold and damp is notoriously common, and the consequences are very serious. Vanity or fashion in the higher ranks, poverty in the lower, and ignorance in both, tend to produce this. The infants of the poor are clothed too little, and exposed to the air too much; whilst the infants of the higher classes are clothed too much, and exposed to the air too little; and the result is, as regards London, that between one fourth and one fifth of all the infants baptized die before they reach the age of two years. The foolish error of hardening children, as it was termed, by exposing them to cold and wet, and by plunging them in cold water even in the winter season, is happily almost obsolete. It was sanctioned by no less a man than Locke, in his treatise on Education, wherein he recommends that boys should wear shoes with holes in them, to allow free ingress and egress to water; the philosopher forgetting that, in such cases, any shoes at all must be considered a superfluity. Dr. Combe alludes to the instructive experiments of Dr. Milne Edwards, shewing, that "the power of producing heat in warm-blooded animals is at its minimum at birth, and increases successively to adult age," and that young animals, so far from being warmer and more capable of resisting cold than older, are actually colder and lose heat more quickly. The practice, already spoken of as enforced by the laws of France, of taking newly-born children to the mayor's office for registration, is perhaps the chief cause of the great mortality of young children in that country. In England the children who are old enough to run about are those who suffer most from injudicious clothing, the upper part of the person being often entirely uncovered. As boys grow older they are protected by warmer clothing, but girls continue to have the neck, and at least half of the chest, quite exposed except when walking out, and even then not adequately defended from cold winds. Very often their clothing is altogether scanty; and bowel complaints, frequent coughs, and enlarged cervical glands, are the common consequences. It is remarked in Dr. Beddoes's tract on consumption, that although the Dutch inhabit a damp climate, and inundate their houses with water for the sake of cleanliness, and have very imperfect floors dividing the upper rooms from the lower, they seldom suffer from colds; and he ascribes this to the warmth of their habitual clothing. If so, the Dutch women may well bear a little ridicule of their rotund figures, and the men are wise to invest themselves, as they do, in almost as many waistcoats as the gravedigger in Hamlet puts off upon our stage, before he begins to dig. Too much clothing may indeed be worn, as well as too little; but if we see people shivering through a winter's day when taking exercise, the direct inference seems to be that they are not sufficiently clothed. Those who can take little exercise generally require warmer clothing than very active persons can bear without oppression.

Of late years we have heard of several instances in which persons long habituated to wearing flannel have been ordered to leave it off, as if it were a most hurtful custom; and of their having done so with impunity. This is one of the ingenious recommendations by which the practitioners of watering-places study to surprise and mystify the pampered invalids who crowd to such places of resort. The patient is generally ordered to leave off his flannel, "for it is killing him:" he is to use the shower-bath of saline water, to walk or ride many miles a day, to eat mutton-chops, highly seasoned, three or four times a day, and to drink sherry as often. Bread alone is to be taken with the meat, or perhaps rice; no vegetables, "they are poison." An amusing book might be written, and we trust that some day one will be written, concerning the practices prevalent in and peculiar to every place which is entitled a spa; places the haunt of many who have no ailment but of the mind and heart, idleness and selfishness; both of which find relief in those communities of sudden growth, in which no man cares for his neighbour, or for the poor; in which most men may escape the trouble of every public and private duty; and in which the hotel-keepers, the trades-people, and above all, we blush to say, too many of the medical men, devote all their energies to the delusion of human folly and the gratification of human weakness.

But for plain honest men who practise among rational people, among residents, whom the pampering and stimulating of a few months cannot dispose of, because they cannot be *sent away* cured, but must stay and relapse within view, we should think the judicious rules laid down by Dr. Combe respecting flannel would be more useful. He justly represents that, being a bad conductor of heat, it prevents that of the body from being too suddenly dissipated, and protects it also from sudden external vicissitudes of heat and cold; whilst its texture gently stimulates the cutaneous vessels and nerves, and readily absorbs the cutaneous exhalations. He allows, at the same time, that to some persons of delicate constitution, and in hot climates, it is hurtful, by irritating the skin and exciting too much perspiration; in which cases fleecy hosiery, if the object is to avoid irritation, or cotton, if the perspiration be excessive, should be substituted for it. Correct views on this point are sometimes of far too much consequence to admit of being trifled with; as where an order to wear or not to wear flannel may affect the health of a fleet or an army.

"The advantages of flannel as a preservative from disease in warm as well as in cold climates, are now so well understood, that in the army and navy its use is urgently, and with great propriety, insisted on. Sir George Ballingall, in his valuable 'Lectures on Military Surgery,' (p. 92,) has some very judicious remarks on the influence of warm clothing in preserving the health of soldiers; and after adducing the testimony of Sir James Macgrigor to show that, in the Peninsula, the best-clothed regiments were generally the most healthy, Sir George adds, that when in India, he had himself a striking proof of the utility of flannel in checking the progress of a most aggravated form of dysentery, in the second battalion of the Royals. Captain Murray, also, late of H. M. S. *Valorous*, told me that he was so strongly impressed, from former experience, with a sense of the efficacy of the protection afforded by the constant use of flannel next the skin, that when, on his arrival in England, in December 1823, after two years' service amid the icebergs on the coast of Labrador, the ship was ordered to sail immediately for the West Indies, he ordered the pnrser to draw two extra flannel shirts and pairs of drawers for each man, and instituted a regular daily inspection to see that they were worn. These precautions were followed by the happiest results: he proceeded to his station, with a crew of 150 men; visited almost every island in the West Indies, and many of the ports in the Gulf of Mexico; and, notwithstanding the sudden transition from extreme climates, returned to England without the loss of a single man, or having any sick on board on his arrival. It would be going too far to ascribe this excellent state of health solely to the use of flannel; but there can be little doubt that the latter was an important element in Captain Murray's success." (P. 87.)

Dr. Combe observes that, in a variable climate like ours, it is hurtful to delay putting on flannel until the winter has fairly set in: its protection is most wanted in the sudden changes from heat to cold which characterize the autumn. It may be added to this remark, that the unwise haste with which many individuals throw off their winter garments in the first warm days of spring is one of the most prolific sources of disease in that insalubrious season. The only safe rule is, that the winter clothing should be worn until it becomes oppressive, which it will not often be before the middle of March, and which it sometimes does not become before the first week of May. Then the sudden heat oppresses the feeble and delicate; some of whom it affects with fever, and all with extreme lassitude, whilst the phthisical, who have struggled through the winter, are swept away with a rapidity which justifies the ancient prejudice existing in England, among the poor, against a warm month of May. In giving directions concerning clothing, these circumstances, and individual peculiarities, should always be taken into consideration.

The families into which Dr. Combe's book finds its way will, we hope, all adopt his advice respecting the proper airing of bed-rooms and bed-clothes. They are generally made up with as much haste as if the general air was infectious. In all weather except damp or very cold weather, the custom of Italy, spoken of by Dr. Combe, might be advantageously adopted; the bed-clothes should be thrown over chairs, the mattresses shaken up, and the windows thrown open for the greater part of the day. In the rooms in which children sleep these measures are still more desirable. He must be eloquent, however, above most physicians, who can persuade mammas to make so great a revolution as to send out their children to walk at the hour when the beds are to be made, and to let the beds be aired well in their absence. The very nurse-maids protest against dressing themselves to walk out at ten o'clock; and the poor children are made to yield to customs hurtful on whichever side they are viewed. The beds are covered up in the coolness of the morning, and the children are taken out in the heat of the day. The closeness of bedrooms and the unventilated bedding are, we doubt not, often the causes of tormenting watchfulness; and of chronic debility in those who repair the broken slumbers of the night by hours taken from the morning. People forget that half of their time is passed in the bed-room; half the time at least of many, and one-third of the time of all; and that to breathe impure air all that time is likely to be injurious. The large size of the rooms or houses of the comfortable classes, combined with good food and clothing, counteracts the evils that would otherwise arise from these causes. Among the poor, who live in crowded rooms, and are ill-fed, the want of ventilation is well known to introduce every form of pestilence.

The salutary influence of the light of the sun is very properly made the subject of observation by Dr. Combe. Like all other influences of this class, it is especially important as regards children. In addition to the instances mentioned by Dr. Combe, it is to be remembered that Dr. Milne Edwards has proved that the privation of light prevents or retards the remarkable transformations undergone by tadpoles; and it is no violent inference that the growth and healthy development of children may be influenced by a cause acting so generally and so powerfully. The size, airiness, and lightness of nurseries is not always sufficiently attended to, even in large houses: the children are immured in cheerless rooms, looking on dark shrubberies, or on the back-yards and chimneys of a town. The poor have no choice of rooms, and sometimes inhabit courts into which the light of the sun can hardly penetrate, and where disease is ever to be found among the squalid children who are not strong enough to run from home.

There are few countries in which the enjoyment of a bath is so difficult to be procured as in England. It would be well if those who are to possess local authority under the new Act of Parliament would read Dr. Combe's excellent remarks on this subject, and would, in each borough in the kingdom, institute warm and cold baths. Nothing would so much conduce to the comfort as well as cleanliness of the workmen, and to the health of all, as the frequent opportunity of bathing in warm or cold water, at a trifling expense. In every school, at least, there should be baths, not only for habitual use, but because, in the maladies of young persons, baths are so often valuable as remedial means, and yet so often pretermitted, because to order a bath is to trouble and disturb the tranquillity of the whole house. The use of the vapour-bath, also, is most beneficial in many constitutions, imparting a power of resisting cold to many who were previously subject to frequent catarrh, and sometimes dissipating an incipient attack of cold or rheumatism, or any other effect of checked perspiration. On all these points Dr. Combe's directions are ample and well considered; and they are concluded with some observations, written with his usual good sense.

"I notice these facts to show that attention to the health of the skin is really influential in preserving the tone of the nervous system, and in contributing to mental and bodily comfort, and not for the purpose of inducing persons in bad health to have recourse to the bath of their own accord; which they ought never to do, as they may chance to suffer from using it unseasonably. No rules of universal application can be laid down, and this is not the place for a professional disquisition.

"If the bath cannot be had at all places, soap and water may be obtained everywhere, and leave no apology for neglecting the skin; or, as already mentioned, if the constitution be delicate, water and vinegar, or water and salt, used daily, form an excellent and safe means of cleansing and gently stimulating the skin: to the invalid they are highly beneficial, when the nature of the indisposition does not render them improper. A rough and rather coarse towel is a very useful auxiliary in such ablutions. Few of those who have steadiness enough to keep up the action of the skin by the above means, and to avoid strong exciting causes, will ever suffer from cold, sore-throats, or similar complaints; while, as a means of restoring health, they are often incalculably serviceable. If one-tenth of the persevering attention and labour bestowed to so much purpose in rubbing down and currying the skins of horses, were bestowed by the human race in keeping themselves in good condition, and a little attention were paid to diet and clothing, colds, nervous diseases, and stomach complaints, would cease to form so large an item in the catalogue of human miseries. Man studies the nature of other animals, and adapts his conduct to their constitution: himself alone he continues ignorant of, and neglects. He considers himself as a being of a superior order, and not subject to the laws of organization which regulate the functions of the inferior animals; but this conclusion is the result of ignorance and pride, and not a just inference from the premises on which it is ostensibly founded." (P. 101.)

With this most excellent advice we must conclude our quotations from this section of Dr. Combe's work, the whole of which will repay the medical reader who peruses it; whilst it is full of good advice for the public. The value of such advice will be estimated by those who know how often a dry harsh state of the skin gives warning of some unnoticed derangement in the health of a child; how often depression of mind is associated in adults with a peculiar state of the surface; and how important attention to the functions of the skin is in the prophylactic, and we may add in the curative, treatment of phthisis pulmonalis.

The admirable chapters on the Muscular System, and on the Effects of, and Rules for, Muscular Exercise, we must pass over without comment; although we hope every parent, and every person concerned in the education of youth, will read them with attention. The parts of the work relating to the Bones and to the Lungs, also, we can only mention with general commendation: the latter, as may be supposed, is full of interest, as it includes the subjects of good or bad, or infected, air,—the effect of different occupations, situations, and arrangements,—and considerations relating to the ventilation of public halls, churches, schools, and houses.

The chapter on the Nervous System and Mental Faculties, and the one which follows it, entitled Rules for Mental Exercise, demand more particular notice, relating as they do to circumstances not unfrequently overlooked by the practitioner, and yet of vital consequence to his patients.

The name of Combe is so associated with the exposition of the doctrines of phrenology, that it must have been difficult for the author, in his brief but expressive account of the brain and its functions, to avoid, as he very judiciously has done, any allusion to the science, excepting those first principles of it which rest on the belief that the brain is the organ of mind, and to the fact that a majority of physiologists consider the anterior lobes as the seat of the intellectual faculties; a belief which the large experience of the reflecting and sagacious Cuvier supported; comparative anatomy having, according to his own statement in the Report to the Institute on the experiments of M. Flourens, constantly offered a confirmation of it, in the proportion of the volume of these lobes to the degree of intelligence of animals. The vague popular notion that the intelligence was something not dependent, even as regarded its development and manifestation, on a material organ, the idea of such dependence being very erroneously considered as a mark of *materialism*, has been an obstacle to the reception of a kind of knowledge which would have shewn the great imprudence of overtasking the brain of young persons, particularly of those exhibiting much intellectual alacrity; of calling upon the young, and those in whom the brain was imperfectly developed, for the performance of intellectual functions, which were with as little reason to be expected as a nice sense of colours in the blind, or of sound in the deaf. Even in the present day, when all profess to see the impropriety of such forcing of the juvenile intellect, we not unfrequently observe lively children distressed and fretted by the vanity of their parents; called upon to read when they are indisposed to read, and to recite when they are disposed to sleep. Dr. Combe, with much justice, condemns the unnatural system by which children are for months consigned to the cheerless labours of school, and then for six weeks permitted to run wild in perfect mental idleness. As is the case with other organs, alternate exercise and rest are required by the brain, but not alternations of extremes. The true ends of education have, indeed, been too much lost sight of, as if the only object to be kept in view had been a certain amount of acquisition in a given time; an error much opposed to a diffusion of the love of knowledge, and the effects of which are exhibited in the immediate abandonment by young men and young women of all intellectual habits, when they are emancipated from school for life. It is, however, but just to say, that, in most schools of modern establishment, more care is taken to ensure health of body and of mind; the continuous application is shortened, the hours of relaxation are more frequent; and we are convinced, from memory and observation, that young people are both healthier and happier under this more enlightened, or, we should say, more natural system, than of old. Under the old *régime*, six or eight hours of each day were passed in bodily inactivity, in a vitiated atmosphere; and this for the sake of going through certain lessons, which would have been much better learned in one-third of the time: the consequences were a depressed and vagrant mind, and very often a feeble frame, unable to resist atmospherical influences; consequences which were only counteracted by the natural propensity of young people to very active exercises when the hour of exercise presented itself; and only thus counteracted in boys, who were not debarred, as too often happened in the case of girls, from the use of their voluntary muscles and organs of voice.

If examples did not daily convince us of the fact, one would not think it possible that intellectual beings would ever be insensible to the good effect of judicious mental exercise upon the body, and to the folly in some cases, and cruelty in others, of placing themselves in a condition, or consigning other individuals to a condition, in which the mind is deprived, for a length of time, or entirely, of proper mental stimuli.

We honour Dr. Combe for his humane mention of the dismal fate of governesses in great families. A young and accomplished female, perhaps the daughter of a clergyman, and once the child of a home of affection, where every liberal accomplishment was valued, goes down to some remote province, or enters some vast mansion in the metropolis, far from every friend, to take the charge of the children of a fashionable mother. She is never admitted to the table of the great man, and, if allowed to beguile some listless hours for the lady of the house, she is taught to fly when the lord of the mansion returns, or to make an ignominious retreat before fashionable visiters. Her apartment is up the back stairs, close to the nursery, where, when her daily round of duties is performed, she has no resource but reading; no society, no companion, no amusements. Her meals are solitary, and the very servants, if insolent, despise, if good-natured, pity, her forlorn condition. Better far, indeed, it would be for her, if she had been brought up with less care, and could partake of the ungenteel mirth of the servants' hall. As it is, the mind preys upon itself, the imagination reigns uncontrolled, and the result is, that few young women ever fill such a situation for six months without becoming invalids. Indigestion, hypochondriasis, nervousness, the loss of colour, and of strength, and of cheerfulness, (all perhaps long unheeded,) mark these unhappy victims. Some of the most pitiable cases of human suffering which we have ever witnessed have occurred in young persons of this class, and which wholly owed their origin to too much solitude, bodily inactivity, and the mental apathy or restlessness produced by a want of cheerful conversation and the ordinary resources of social intercourse.

The retired man of business seems less an object of commiseration; but his sufferings are soon almost equally severe: his mind suffers from want of exercise; he grows apathetic, and breaks up rapidly, amidst the wonder of his neighbours. Thus, the man oppressed with business flies for relief to indolence, and finds his old evils only exchanged for other evils. True wisdom should be exercised in so proportioning exercise and rest as that one may relieve the other; and the powers of the mind, exercised without being too much excited or too much fatigued, may then last as long as the powers of the body. We have occasionally heard the testimony of soldiers who have been subjected to solitary confinement, and, although they were persons of small habitual intellectual activity, they always spoke of it with extreme repugnance, looking upon it as even worse than the degrading punishment of flogging. The effects of what is called the silent system of treatment in the American prisons cannot but be attended with much mental torture, and even disease. By the healthful exercise of the mind induced by the necessity of exertion, the advantages of fortune are compensated for in all the middle and lower ranks of society: when freed from this necessity, indolence brings in its train irritability, caprice, nervous disorders, untranquil digestion, imperfect sleep, and sometimes mental derangement. In the large prisons of London, in which the wretched inmates enjoy no relaxations but such as are vicious, we have been struck with the rapid change effected upon them: the countenance soon loses not its vivacity only, but its intellectual expression, and eccentricities appear which border on madness. The whole of this subject, which Dr. Combe had before very ably treated of in his work on Mental Derangement, is introduced in the most interesting manner in his present publication; and his remarks will, we trust, meet the eye, and awaken the mind, of many a listless reader, male and female, and, by explaining to them the cause of the mental languor and discontent which overpowers them, rouse them to the exertions by which such demons are best resisted.

But, in this age of exertion and ambition, there are innumerable victims of *too great* an exertion and excitement of the brain. Every man's observation must have shewn him such; and they abound in our own profession. The most moving persuasions are less effectual in checking such mental excess than facts of the following striking kind. After illustrating the effects of irritation of the brain by what takes place in the eye when over-exercised, Dr. Combe adds:

"Precisely analogous phenomena occur, when, from intense mental excitement, the brain is kept long in a state of excessive activity. The only difference is, that we can always see what happens in the eye, but rarely what takes place in the brain. Occasionally, however, cases of fracture of the skull occur, in which, from part of the bone being removed, we can see the quickened circulation in the vessels of the brain, as easily as those in the eye. Sir Astley Cooper had a young gentleman brought to him, who had lost a portion of his skull just above the eyebrow. 'On examining the head,' says Sir Astley, 'I distinctly saw the pulsation of the brain was regular and slow; but at this time he was agitated by some opposition to his wishes, and directly *the blood was sent with increased force to the brain, the pulsations became frequent and violent: if, therefore,'* continued Sir Astley, '*you omit to keep the mind free from agitation, your other means will be unavailing*'' in the treatment of injuries of the brain. A still more remarkable case is mentioned by Dr. Caldwell, as having occurred to Dr. Pierquin, in the hospital of Montpelier, in 1821. 'The subject of it was a female, at the age of twenty-six, who had lost a large portion of her scalp, skull-bone, and dura mater, in a neglected state of lues venerea. A corresponding portion of her brain was consequently bare, and subject to inspection. When she was in a *dreamless sleep*, her brain was *motionless*, and *lay within* the cranium. When her sleep was *imperfect*, and she was agitated by dreams, her brain *moved* and *protruded without* the cranium, forming *cerebral hernia*. In *vivid* dreams, reported as such by herself, the protrusion was considerable; and when she was perfectly awake, especially if engaged in active thought or sprightly conversation, it was still greater " This protrusion arose, of course, from the greater quantity of blood sent to the brain, during its activity, than when it was quiet; and, if the case be accurately reported by Dr. Pierquin, it is certainly one of the most interesting on record.

" We are conscious, indeed, of a flow of blood to the head when we think intently, or are roused by passion; and the distention of the small vessels of the brain is not the less real or influential on account of its being hidden from our view. Too often it reveals itself by its effects when least expected, and leaves traces after death which are but too legible. How many public men, like Whitbread, Romilly, Castlereagh, and Canning, urged on by ambition or natural eagerness of mind, have been suddenly arrested in their career, by the inordinate action of the brain induced by incessant toil! and how many more have had their mental power for ever impaired by similar excess! When tasked beyond its strength, the eye becomes insensible to light, and no longer conveys any impressions to the mind. In like manner, the brain, when much exhausted, becomes incapable of thought, and consciousness is almost lost in a feeling of utter confusion." (P. 287.)

These are truths well worth remembering, and they are plainly and well expressed. Medical men might sometimes save valuable lives by attention to these circumstances; and by representing, supported by allusions to facts of this kind, brought before persons regardless of all common cautions, that by too great anxiety to do much, they were actually defeating their own ends; overleaping themselves, to "fall on t'other side."

In children, also, there is not only abundance of proof that too much exercise of the mind aggravates the disposition to some diseases of the brain, but much reason to think that such over-exertion creates such a disposition in a healthy brain, and, by enfeebling the whole body, occasionally brings on the whole train of affections called scrofulous. Dr. Combe refers to a sensible work by Dr. Brigham, published at Boston, in America, in 1833, in which it is stated that, in the passion for making prodigies of the infant citizens of the States, their parents are supplied with "INFANT Manuals of Botany, Geometry, and Astronomy;" and he relates some striking instances of the destructive effects of this mental forcing. We do not consider these practices, and their results, as constituting any arguments against infant schools, which, when well conducted, and chiefly with a view of disciplining the tempers of the little scholars, and keeping them from quarrels and mischief, are among the most valuable of modern institutions. If much is attempted beyond this, the result is generally disastrous: the child grows up, remarkable for its acquirement and for its extreme sensibility; the frame is delicate or sickly, and, when at length he is expected to come forth and challenge the public regards, he fades away before the eyes of his distracted parents, and sinks into the grave.

Among the students of colleges where prizes are to be contested for, or honours gained, an erroneous kind of reasoning prevails, that, if the faculties can accomplish a certain quantity of work in eight hours, they can accomplish double the quantity in sixteen. The late Dr. Gregory, of Edinburgh, whom no one could suspect of being the apologist of idleness, used in his lectures to demonstrate the fallacy of this reasoning, and the dangers of it; which, after all, it was observable that many of his hearers incurred. As a dissuasive from too much night-reading, he used to mention that being himself anxious when a student to do a great deal in a short time, he acquired the habit of sitting up a great part of the night; and that, as he was accustomed to mark the passages he read, he was surprised to find that in the morning he was often unable to recollect reading the passages which he had so marked. A better illustration could not have been given of the fruitlessness of such night-work, than its effects on the mind of so vigorous and abstemious a person. The delusive notion that the mind can be most advantageously employed at night is disproved by many instances, and by those of two persons alluded to by Dr. Combe, whose intellectual fame might indeed satisfy the largest ambition. It was Sir Walter Scott's practice to write in the morning; and he gave the rest of the day to business or amusements. Mr. Southey, one of the most industrious and learned of living writers, is, we believe, always in his study at an early hour.

The impression made upon us towards the end of the college session in Edinburgh, by the unhealthy aspect of our fellow-students, has not yet left our memory; and further experience has established these results of over-study by many lamentable instances: debility, palpitation of the heart, dyspepsia, fever, consumption, and insanity, have, we think, been clearly observed by us to arise, and some of them frequently, from intemperate application to study, in students of our own profession. Every other profession could furnish parallel examples. That of Sir Humphrey

Davy is introduced by Dr. Combe: a dangerous fever, and long continued debility both of body and mind having been incurred by that illustrious philosopher when lecturing at the Royal Institution, in consequence of intense mental exertion. The biography of many an accomplished youth, snatched away by death when he seemed advancing to fame, attests the existence of similar imprudences followed by similar consequences at our great English universities. And all these evils arise from forgetting, apparently, that the mind depends for its manifestation on the brain, a bodily organ, which cannot be kept in health without some rest, and without general exercise of the body, good air, proper food, and diversified employment. To use language quoted by Dr. Combe from the American Annals of Education, and there applied to the case of a young clergyman, the duties of the mind and heart are done, and faithfully, in many of these instances, but "three hundred and seventy-five muscles, organs of motion, have been robbed of their appropriate action for nine or ten years, and now they have become, alike with the rest of the frame, the prey of near one hundred and fifty diseased and irritable nerves."

We have often thought it would be interesting to collect the examples of men of intellectual habits whose lives have terminated by paralysis. Perhaps in some cases this is to them only a form of natural death,—death beginning at the brain. But in countless cases it arises from too much mental exertion or anxiety, conjoined with sedentary habits, and too great indulgence at table. Yet as age advances, every man should moderate his expectations with respect to the productions of the brain; and it is no unworthy study to preserve its power co-equal with the powers by which mere existence is prolonged. How wise as well as beautiful was the advice of Cicero, who, insisting strenuously for the continuance of mind in the oldest men, if industry remained, yet adds, "habenda ratio valetudinis; utendum exercitationibus modicis; tantum cibi et potionis adhibendum, ut reficiantur vires, non opprimantur: nec vero corpori solum subveniendum est, sed menti, atque adeo multo magis, nam hæc quoque, nisi tanquam lumini oleum instilles, exstinguitur senectute!"

Dr. Combe's observations on mental exercise are characterized by his usual good sense and copiousness of illustration: and indeed, of his whole work, we are justified in saying that it is one which practitioners may with great propriety recommend to their educated patients; who, in proportion to the sound knowledge they are persuaded to acquire, will always be found more obedient to judicious medical advisers, less capricious, and more likely to do credit to those whom they consult.

The works of Dr. Dunglison and Dr. Kilgour are rather addressed to medical readers than to the public, the first being intended as a text-book for lectures on Hygiène in the University of Maryland, and the second published in the form of lectures as part of a course on Therapeutics. The lectures of Dr. Kilgour chiefly relate to private hygiène; Dr. Dunglison's book comprehends much that relates to hygiène as respects communities. Dr. Kilgour's style is however so lively and colloquial as irresistibly to recommend it to the general reader; and the variety of matters in Dr. Dunglison's, interesting to official persons as well as to individuals, give it a title to careful perusal much beyond the limits of the medical profession.

To Dr. Dunglison's text-book is prefixed a short physiological proem, in which, after defining the object of Hygiène to be to enquire into the circumstances which produce disease, or into the influence of physical and moral agents on healthy man, and thence to deduce the best means for preserving health, and for developing all the healthful energy of which the functions are capable, he proceeds to notice several points of general physiology, necessary for the perfect comprehension of the subject. The topics then treated of are Atmosphere and Locality, Food, or the Materia Alimentaria, Clothing, Bathing, Exercise, Sleep, Corporeal and Mental Occupations; and there is a supplementary chapter containing many facts touching Malaria and Temperature. In all these chapters it is evident that the author is a physician of extensive acquirements, literary and professional, and possessed of that good sense which is so essential to the proper consideration of some of the branches of knowledge which have engaged his attention. This is very conspicuous in the large portion of his book devoted to the consideration of Diet; and his opportunities of seeing the effects of different modes of life in both the New and the Old World have enabled him to correct some of the hasty conclusions of the European writers. In the chapter on Corporeal and Mental Occupations he controverts an opinion which has during late years found some supporters, that the pursuit of letters is unfavorable to longevity; and shews the error of attributing the deaths of many of the poets who died early, to the exercise of their imagination, when there is such great reason to ascribe their premature decline to irregularities of life:—these irregularities, however, it should have been remembered, are much associated with the imaginative temperament. In an excellent treatise on Physical Education by Dr. Caldwell, an American physician, it is stated that of the fifty-six delegates who signed the Declaration of Independence, almost all of whom were men of well-regulated and active minds, two died from accidents; and the aggregate years of the remaining fifty-four were 3609, giving to each an average of sixty-six years and nine months. The average duration of life in twenty mathematicians, taken promiscuously, was seventy-five years. That of an equal number of poets was only fifty-seven. These facts, cited by Dr. Combe, furnish a useful lesson. The mind seems recreated and the body strengthened by diversified mental occupation, and, fortunately, this is exactly what every man's private and public duties impose upon him. To neglect either is to incur some kind of penalty.

The first portion of Dr. Dunglison's book, or that relating to Atmosphere and Locality, contains much to which the medical reader will refer with interest. In his first chapter he examines the influence of the density and temperature of the atmosphere on the human body, and adduces instances in proof of the effects of mere density having been much over-rated. He refers to the fact of the residents of the farm of Antisana in Quito suffering no inconvenience from its atmosphere, after a short time, although its elevation is 13,400 feet. Cassini maintained that no animal could exist at 15,640 feet; but the commissioners sent to measure the earth at the equator lived for a considerable time on the summit of Pichinca, 15,939 feet above the level of the sea; and whilst there, often saw the vulture soaring a thousand feet above them. The opinion rested on the presumption that at the height of 15,640 feet the atmosphere is one half rarer than at the level of the ocean, and "on the fact, that if the air be suddenly dilated one half under the receiver of the air-pump, an animal placed under it dies." Such, observes Dr. Dunglison, might be the effect if the density were suddenly diminished, but man seems endowed with a remarkable capability of resistance to such influences when gradually exerted, or even when as rapidly exerted as in the ascent of a balloon to more than 20,000 feet. The elevated regions of Asia afford examples of a mild climate at great heights above the sea, as on the crest of the Huketo pass, and on Zinchen, the first more than 15,000, the second more than 16,000 feet high. In these prodigiously elevated localities, the climate is pleasant, horses are numerous, kites and eagles fly about, and small birds and locusts abound. At 13,600 feet, were fields of barley and turnips; and a little lower, thyme, sage, juniper, sweetbriar, and gooseberries; and even vineyards and groves of apricots. At Nako, in the midst of the Himala range, 12,000 feet above the sea, the grain was yellow in August; and there was a broad sheet of water, surrounded by tall poplar, juniper, and willow trees. Yet, observes Dr. Dunglison, the latest French writers on hygiène copy from their predecessors, and state that at 12,790 feet (English) no trees are found, and that at 14,708 feet there is no trace of vegetation.

"Even the sanitary depôts, for those suffering under the diseases of the lower and hotter parts of India, are situated, in some instances, higher than the point assigned by Londe as the limit to human salubrity. Dargeeling, in the Sikkim mountains, 330 miles from Calcutta, has been recommended as a sanitarium. Its height is about 7,218 feet above Calcutta, and its mean temperature is calculated to be 24° below that of Calcutta, and only two degrees above that of London. A convalescent retreat has also been provided at Simla, a station among the hills between the Suttledge and Jumna, near Sabhatto, and 7,500 feet above the level of the sea." (P. 44.

Dr. Dunglison subsequently enters on the examination of the effects of temperature, deducing from numerous facts the conclusion, that the temperature of the body may be lowered beyond what is natural to it, nearly twice as much as it can be raised, consistently with life. It appears, he observes, that independently of all other considerations, the elevated temperature of the torrid regions of the globe is positively detrimental to animal health; the constant evaporation by the cutaneous and pulmonary transpiration maintaining the absorbents of the intestines in a state of irregular erethism, and hence disposed to assume a morbid condition; in which way he would explain not only the frequent occurrence of diarrhœa, dysentery, and cholera, but the diseases of the liver which so universally are found to attend inflammation of the upper part of the intestinal canal in those climates. The method of enlarging the liver of geese for the famous Strasburg *pâtés* is by nailing the unfortunate birds to a plank by the web of the foot, near a large fire; abundance of food is given, and they are kept from drink. Such is the manner in which man continues to shew his superiority over the lower animals! In many of the unhealthy districts of India, dogs are said to be subject to the endemic diseases of the climate. Experiments on animals have proved that, when they are exposed to high temperature, they consume less oxygen during respiration: the extreme arterial vessels seem deprived of their usual energy, and the arterial blood flows on, little changed, into the veins; effects which are perhaps to be referred to a diminished energy of the brain and nervous system; many other effects of which are observable in those who have been long in hot climates. Such climates are especially unfavorable to those disposed to cerebral diseases: Dr. Dunglison says he has met with cases of hemiplegia in young men, between twenty and thirty years of age, developed by a short residence in India. In opposition to the opinion of M. Rostan, who is certainly prone to take up opinions very hastily, and who asserts that warm climates are beneficial to the scrofulous, Dr. Dunglison refers to the testimony of Sir Whitelaw Ainslie, who observes, that perhaps of all disorders, that to which the climate of India proves most *un*genial is scrofula: indeed, that experienced physician goes so far as to say that he never knew one individual in India, who was of such a constitution, and remained in tolerable health for ten months together. Soldiers of a scrofulous constitution become affected, he says, with "frightful and ravaging ulcers," and "are fit for nothing but lumbering up an hospital." Gout and rheumatism are less prevalent and less severe in hot climates, and consumption is rare.

Every one knows that the first effect of a moderately depressed temperature is agreeable and exciting: all the functions are increased, except that of cutaneous transpiration, the diminution of which is compensated for by an increase of the urinary secretion. When subjected to a temperature of between thirty and forty-five degrees of Fahrenheit, or lower, the diminished cutaneous exhalation and the depressed circulation lead to engorgement of the air-tubes, producing bronchitis, winter cough, &c. The fatal effects of a severe frost on old people was observed by Dr. Heberden; and Dr. Beddoes found that, among persons above sixty years of age, the greatest number of deaths took place in the coldest months, and the fewest in the middle of summer. But Dr. Dunglison remarks, that it is only the first part of this observation which accords with their experience in Virginia; excessive heat proving fatal to many elderly persons, by occasioning disorders of the lining membrane of the intestinal canal.

At a still lower temperature, the nervous system becomes torpid, and an intense desire for sleep succeeds. The well-known instance of Dr. Solander does not require to be quoted. Dr. Dunglison alludes to still more memorable examples during the retreat of the French army in Moscow, in 1812. In two of the nights of December, the thermometer was as low as twenty-seven and thirty-two degrees below zero of Fahrenheit; and in this extreme cold many of the horses died, and the soldiers, who were without furs and cloaks, were struck with stupor if they took the least rest; death being preceded by pallor of the countenance, a kind of idiocy, difficulty of articulation, defective vision, and sometimes a total loss of sense. Supported by their comrades, they would stagger on in this condition until they fell down dead.

In such instances as the last, the effects are plainly to be ascribed to cold alone, acting with an intensity which animal life could not resist. But there is an evident difficulty in separating the effect of mere temperature from those produced by combinations of certain degrees of heat with other causes, when speaking of the diseases peculiar to different climates. The hygrometric states of the atmosphere, for instance, always require to be taken into calculation. To the consideration of this subject, as well as of the atmospheric vicissitudes which so powerfully affect the frame, and the effects of electricity and light on the functions, Dr. Dunglison has devoted a section of his first chapter. The following extract comprehends the principal facts mentioned in relation to the influence of moisture.

"The barometric and thermometric influences of the air are exerted with more or less energy upon the animal system, according as its hygrometric condition is more or less considerable, that is, according as it is dry or damp. Dry air, for example, is heavier than moist, inasmuch as watery vapour is lighter than air in the proportion of .625 to 1,000. When the air, consequently, is largely charged with moisture, the mercury in the barometer falls; and, on the other hand, when it is dry, the mercury rises. We have seen, again, that the sensations of heat and cold, experienced from the air, are greater when the air is damp, owing to the presence of water between its particles adding to its conducting power; and, lastly, that as the dissolving power of the air augments in proportion to its dryness, and temperature; its action upon the fluids of the body must be less in a moist than in a dry atmosphere.

"It may be remarked, by the way, that a moist atmosphere is better adapted than a dry one to dissolve various animal, vegetable, or mineral substances, which are susceptible of volatilization. We have many instances to prove, that volatilizable substances are sooner converted into the gaseous state under such circumstances. Lime-burners are well aware, that limestone can be burnt, and reduced to the state of quicklime, much sooner in moist than in dry weather; and, in the latter case, they not unfrequently place a pan of water in the ash pit, the light vapour of which,—lighter, as we have seen, than atmospheric air,—assists in carrying off the carbonic acid gas, which is heavier. Camphor is found to volatilize with much greater celerity in damp situations, and every one has noticed the fragrance of a garden after a summer's shower. There are certain bodies, too, which require the presence of moisture for their escape;—thus, the odorous particles of argillaceous substances are quiescent until they are breathed upon, or, in other words, become moistened by the fluid from the lungs, or by moisture of some kind, after which the mineralogist readily recognizes their characteristic odour. Every one must have noticed how powerfully the stench of putrid ditches is conveyed to the olfactory organs in summer, previous to rain, when the air becomes charged with moisture, and how readily offensive substances are detected in a fog by the same sense.

"The agency of moisture is doubtless also concerned in the conveyance of various emanations from the soil, which produce endemic disease. It has long been noticed, that, whilst the inhabitants of a plain, on the level of a marshy land, have escaped diseases that are known to be produced by the emanations from such land, or by *malaria*,—as it has been termed by the Italians,—those dwelling on neighbouring elevations have suffered extensively. Observation would seem to have shewn, that this malaria is somewhat heavier than atmospheric air, but as watery vapour is incessantly exhaled from the surface of the earth under the influence of solar heat, and as this vapour possesses so little specific gravity, it takes up the heavier miasmata along with it, and, under favourable circumstances, they are deposited on the elevations.

"Similar remarks apply to the communication of the matter of contagion, which would appear to be modified in its activity, by the degree of moisture in the atmosphere, influencing its solubility and volatility; but on this topic our evidence is not quite as satisfactory. The same may be said of epidemic influences, of which our ignorance is unhappily so profound. It may be remarked, however, as some corroboration of this view, that the Harmattan, a wind which blows periodically from the interior of Africa towards the Atlantic ocean, and which is characterized by its extreme dryness, is asserted to put an end to all epidemic and contagious affections,—even to small pox; and it is said that, at such times, the disease is not easily communicable by art.

"We shall find hereafter, that humidity modifies the action of atmospheric electricity on the animal body, as well as the electrical condition of the body itself." (P. 66.)

Dr. Dunglison's remarks on the effects of atmospherical vicissitudes, and on the influence of electricity and light, contain useful information, conveyed in a very unpretending manner, but nothing which is not generally known to medical readers. In the next section, after detailing the consequences of confinement in vitiated air, he treats of the important subject of malaria, or of terrestrial emanations. Observing that the malignant cholera attacked several of the towns of America in the most virulent manner, whilst others, and some to all appearance similarly circumstanced, wholly escaped,—a course accordant with European experience,—he concludes that the complaint required a combination of atmospheric and local causes to induce it, or, in other words, that the causes were of an *endemico-epidemic* character. After noticing similar circumstances, equally inexplicable, respecting typhous fevers and intermittents, Dr. Dunglison dwells at some length on the causes which have been assigned for the terrestrial emanations which unquestionably take place from marshy and other districts; and the conclusions at which he arrives, in which he would himself allow that nothing is concluded, are expressed in the following summary:

"What then is this malaria—arising so frequently from marshy situations as to be called *marsh poison*, but emanating also, at times, from soils far distant from any marshy lands; affecting the whole of our country below tide-water, and more or less unknown in many of our mountain regions; occurring in certain localities in spite of every care, and not producible in others by any process with which we are acquainted? We have endeavoured to prove, that it is not caused, as far as we know, by any ordinary kind of decomposition; that it is not animal in its nature, nor vegetable, nor compounded of both, but that in marshy and stagnant situations it requires, that the bottom, previously submerged, should be exposed to the solar heat. Dr. Ferguson, indeed, considers that a highly advanced stage of the drying process is necessary for its production; and he adds that, in the present state of our knowledge, we can no more tell what that precise stage may be, or what that poison actually is, the development of which must be ever varying, according to circumstances of temperature, moisture, elevation, perflation, aspect, texture, and depth of soil, than we can define and describe those vapours that generate typhous fever, small-pox, and other diseases.

"Such is the negative opinion of Dr. Ferguson with regard to the origin of malaria. On the other hand, Julia ascribes it to a union of *animal and vegetable* putrefactions, but expresses his total ignorance of the nature of the emanation. Dr. Macculloch maintains that putrefaction, in the proper sense of the term, is not necessary, but that the stage or mode of *vegetable decomposition*, required for the production of the malaria, is different from that which generates a fetid gas. Others have supposed the miasm to be animalcular, and others, again, that it is produced by animalcular putrefaction. Dr. Caldwell, in his Prize Essay on Malaria, affirms it to arise from vegetable and animal matter, more especially the former, in a state of "*dissolution.*" "I say dissolution, not putrefaction, because there is good reason to doubt whether that process, in the technical meaning of the term, be necessary to the result. Bilious fever, in all its varieties of type and degree, often prevails in places, where no putrefaction is discoverable. But dissolution, by which I mean the decomposition of dead organic substances, and the reunion of their elements, producing new compounds, is present. *In no other way can the Malaria be formed!'* Lastly, Dr. James Johnson, in a recent work already cited, thinks we are pretty safe in concluding, that, 'generally speaking, it is the product of animal and vegetable decomposition by means of heat and moisture.' Yet, in another page, when speaking of *pellagra*—a singular cutaneous and nervous affection, endemic in the Lombardo—Venetian plains—he expressed himself in a manner, which would seem to shew that he by no means esteemed it 'safe' to deduce any such conclusion; for he wisely observes,—'The cause of this frightful endemic pellagra, has engaged the pens of many learned doctors. But it is just as inscrutable as the causes of hepatitis on the coast of Coromandel, elephantiasis in Malabar, beriberi in Ceylon, Barbadoes Leg in the Antilles, goître among the Alps, the plica in Poland, cretinism in the Vallais, or *malaria in the Campagna di Roma.* It is an emanation from the soil; but whether conveyed in the air we breathe, the food we eat, or the water we drink, is unknown. If this, or any of the endemics which I have mentioned, depended on the filth or dirty habits of the people, we ought to have similar complaints in Sion, or the Jews' Quarter in Rome, the narrow lanes of Naples, and the stinking alleys of all Italian towns and cities. But such is not the case. The Jews' Quarter in Rome is the dirtiest, and the healthiest spot in that famous city. The inhabitants of Fondi, Itri, and other wretched villages in the Neapolitan dominions are eaten up with dirt, starvation, and malaria; but no pellagra, no elephantiasis, no goître, no cretinism, is to be seen. The inevitable and the rational inference is, that each country, where peculiar or endemic maladies prevail, produces them, from some hidden source, which human knowledge has not yet been able to penetrate."

"Such inference, we would unhesitatingly say, is applicable to malaria as we have been considering it; and this is strikingly confirmed by the discrepancies in the opinions of the writers whom we have cited. Can we then, in the state of ignorance that envelopes us, fix positively, or even with any thing like probability, upon any cause, or combination of causes of any kind, likely to give origin to malarious emanation?

"It has been already asserted, that we are uninformed regarding the nature of the emanations from even the most unhealthy situations, where we *know*, from the results, that such emanations exist. They have utterly defied the art of the chemical analyst. They cannot consist of hydrogen, or of carburetted, or sulphuretted, or phosphuretted hydrogen, for no such adventitious gases have been detected by the chemist, which they could readily have been, if present; nor has there been found any additional quantity of carbonic acid gas, or of azote. The revival of the ancient theory of animalcules scarcely requires a comment. It sufficiently shews the obscurity, that environs the subject.

"Such is our ignorance of the nature and causes of the malaria, which emanates from marshy lands more especially—of that which gives rise to remittent and intermittent fevers. But, although unacquainted with it in these particulars, we do know some of the laws by which it is governed." (P. 117.)

Among these laws it seems to be ascertained that, by reason of its specific gravity, it is during the night in greatest concentration near the surface of the earth, so that the inhabitants of the lower stories of houses are most exposed to its agency. But, if a man build his house on a hill-top, thither also may malaria pursue him; for the buoyant aqueous vapour during the day carries up the heavier noxious exhalation. A high wall, or barricade, or an intervening wood, may be a protection against it; and, in several situations near the Pontine marshes, trees having been cut down or forests cleared, fevers and other affections, from which such places were free, have made their appearance. The occasional prevalence of malarious diseases upon heights in the vicinity of marshes seems explained by the raising of the heavier miasmata with the lighter vapours, as above mentioned. But, although in many cases there is no difficulty in supporting the accusation of insalubrity under which marshes must be said to lie in all parts of the world, the malaria is yet too insidious an enemy to be avoided by simple precautions taken against such convicted districts. It assails the loiterer in the loveliest portions of Italy; a country of which the late Dr. Macculloch strongly says, "its fragrant breezes are poison; the dews of its summer evenings are death." There are beautiful districts also in America, Dr. Dunglison informs us, —places where many years of immunity from fevers had given security to the inhabitants, but from which this invisible destroyer, the pestilence which walketh in darkness, has sometimes capriciously driven away the occupiers, and where, after a temporary desolation, it has again left the localities salubrious. And, as to the character of the soil most productive of these noxious influences, all seems yet to be discovered. "It *may*," says Dr. Dunglison, "require an admixture of argillaceous earth. It *may* require animal and vegetable remains. It *may* be a gaseous emanation. It *may*, as Foderé thinks, resemble the product of organic decomposition. All these are possibilities, but requiring substantiation, and in which the negative evidence preponderates largely over the positive." This is not very satisfactory; but Dr. Dunglison is likely to be a better teacher for being suspended in these philosophical doubts, than if he came forth in the character of a champion for any one of these probabilities, to the utter and scornful exclusion of all the rest.

Dr. Dunglison has not noticed Dr. Prout's remarkable observation respecting the slight increase in the weight of the atmosphere which preceded the appearance of cholera in London, in 1832; a fact which, in so hidden a subject, would seem to constitute at least one step to a better acquaintance with one of the worst as well as most mysterious of the enemies to human health.

Looking back upon the "old country," Dr. Dunglison seems to be a little sceptical as regards some of the results stated in the Population Returns respecting the rate of mortality among us: that, for instance, which sets forth the mean annual mortality of

England and Wales as being only 1 in 58; that of the Pays du Vaud, according to Dr. Hawkins, being 1 in 49; of Sweden and Holland, 1 in 48; of Russia, 1 in 41; of France, 1 in 40; of Austria, 1 in 38; of Prussia and Naples, 1 in 33 to 35; and of South America, 1 in 30. The same rate of mortality, he says, is assigned to the United States as that of France, namely, 1 in 40; but he adds, that there can be no authority for this, as the census taken every ten years throughout the United States is deficient in that kind of information. A writer in the American Almanac estimates the mortality in the United States as 1 in 50; yet Dr. Dunglison thinks that the climate in many of the mountain districts equals, if it does not exceed, the mean of England. America, however, is in bad odour with our English insurance offices. Dr. Dunglison was himself obliged to sacrifice a policy of insurance on going out to the university of Virginia, on account of its being required of him to double the premium. Another professor in the same university, wishing to effect an insurance at another office, was told that they must decline insuring the life of any resident of a country in which the rivers were frozen over in a single night! The rate of mortality, he adds, at Philadelphia, is less than that of any European city of which the medical statistics have been taken. We are sorry to perceive that an instance of exaggeration seems to have occurred in the Cyclopædia of Practical Medicine, in the article MALARIA AND MIASMA: its respectable author, Dr. Brown, must, without doubt, have been led into error when he wrote the passage alluded to, in which it is stated that, in the marshy districts of Egypt, Georgia, and Virginia, the extreme of life is forty; and that, at Petersburg in Virginia, (on the authority of Dr. Jackson,) a native and permanent inhabitant rarely reaches the age of *twenty-eight*.

On many points connected with medical statistics and climate, we might transfer from Dr. Dunglison's pages highly interesting observations and facts, and he generally exercises a very sound judgment where discordant opinions have existed among previous writers. The hygienic cautions scattered through his work are useful and judicious, and we do not know so complete a text-book of hygiène as that which he has prepared. We have already spoken in commendation of the dietetic portion of his work, and have only to add, that the chapters on Clothing, Bathing, and Exercise contain numerous particulars interesting to the medical practitioner and to the public. Every subject seems well considered; nothing is neglected, and nothing is pushed to extravagance.

The subjects comprised in Dr. KILGOUR's Lectures are the same, or nearly the same, as those treated of by Dr. Dunglison; but Dr. Kilgour's manner of considering them gives them almost the air of novelty. After a most spirited introduction, to which we shall yet have to refer, he proceeds to speak of the properties of the atmosphere, and of the means of correcting them; such as draining or irrigating the soil, cultivation, rearing and cutting down trees; and then offers much salutary advice to the inhabitants of towns and villages, concerning the preservation of individual health and the health of the community. There is, in the questions noticed in these short chapters, much that is of great consequence, not only to the placid burghers of our own secure towns, but to those who are driven by calamity, or allured by speculation, to new settlements, in which, if any where, a knowledge of the general causes of health and disease is the most valuable knowledge that men can possess, because without it the chances are very great that life will be speedily sacrificed. That the neighbourhood of marshes is more unwholesome than the banks of running rivers is, one would suppose, a truth pretty widely disseminated; but, when we observe the ill-selected site of new habitations in our own country, where every diversity of situation is offered to the choice, we can readily conceive how circumstances, otherwise apparently advantageous, render colonists careless of fixing on a proper ground on which to build. The cultivation of a country is known to improve the climate; the explanation perhaps being, as stated by Dr. Kilgour, that vegetable life is the conversion of certain gases, oxygen, hydrogen, and azote, and carbonic acid, into solid matter; which alteration of bodies from a rarer to a denser state is accompanied with the extrication of heat. There are few things more important to a settler than the rearing or cutting down of trees. Even in England this is far from sufficiently considered with reference to health. Trees, we have seen, may be useful as screens against malaria; they may also be prejudicial, by preventing a due access of air and sunshine. If too closely planted, so as to create a dense shade, in which a heap of leaves is accumulated and undergoing decomposition, the air becomes sensibly disagreeable and damp; and there is no doubt that in such a state it becomes noxious. Although the clearing and cultivation of a new country eventually improves its climate, it is too well known, and has been too dearly learned, that the first effect is very insalubrious. According to Dr. Kilgour's explanation, the miasma, confined before, and for ages, escapes in full force, "and for years after, as the rich soil is ploughed up, it steams forth the deadly air."

Dr. Kilgour makes many sensible remarks on the building of houses; on the situation of houses, the materials chosen for building, and the distribution and size of the apartments. In considering these arrangements, the thing least remembered, generally speaking, is the effect upon the health of those who are to inhabit the new building: it may be reflected upon a little in the choice of the situation, but is often quite overlooked in the arrangement of the interior. The observation of Dr. Kilgour is very just, that the physician has often to regret the confined bedroom in which his patient is placed; and this is sometimes the case in large houses, in which the younger people are consigned to small bedrooms, little better than closets, for ten hours out of every twenty-four.

Any one who walks out of the country into a well-built town at night, must be sensible of the superior warmth of the atmosphere into which he comes. The freer ventilation of a country house, and its isolated situation, are accompanied with the inconvenience of greater coldness, and sometimes of greater dampness. The chief inconvenience of towns is deficient ventilation, added to which, the streets are seldom kept so clear as they might be from decaying refuse matters. The inferior streets, even of towns reputed very clean, are generally damp, and often offensive; and the courts are almost always full of disgusting nuisances, which invite every disease that floats in the atmosphere. The state of the drainage is still very imperfect in many towns, and wherever such a state is most observable, we are quite convinced that our common fevers, in their worst form, are most prevalent. There are many country towns in England in which fever rarely appears, and in which, when it does occur, it is almost invariably in the cottages of the poor, and in ill-drained courts or rows of houses. There are many towns in which one or two cases of malignant cholera appeared in 1832, and exclusively in houses surrounded with nuisances. At this very time, the English cholera, dysentery, and the measles, prevail in several situations, not exclusively among the poor, but much more among them than in the more comfortable classes; and both the measles and dysentery have been in several instances fatal, still chiefly, and for a time entirely among the poor. The state of some of the cottages let to the poor in many courts and alleys of all towns is such as would interdict, under a good police of health, their being let at all; and such houses and neighbourhoods, Dr. Kilgour truly observes, are much more detrimental to the community than the smoke of a manufactory. The state of the parish-church is sometimes, in our opinion, well worthy of more attention. Its want of thorough ventilation, the closed windows, the dark damp corners, the neglected floors and foundations, added to the extraordinary custom of burying dead bodies in the aisles or under the pews, renders the atmosphere almost insupportable to delicate persons; producing faintness, tremors, and nervousness; and, we have often suspected, even more serious consequences. As regards the police of towns, we quite agree with Dr. Kilgour in wishing it extended to regulating the width of streets, and, if courts must be, of courts.

"We have now no walled towns, nor need of them. All streets, therefore, should be of a certain specified width. No dwelling houses should be allowed to be built in courts or alleys. It ought to be compulsory on persons opening new streets to have them running in a direct line. Every house ought to have as much vacant ground behind it as the breadth of the street before it. The houses ought to be all of one height, and built in one line. All pools and stagnant waters ought to be contracted, drained, and covered, at the expense of the proprietors. They are injurious in themselves and a receptacle for every species of filth. As the dwelling houses of the poor may become as much the source of disease as stagnant water, or filth on the streets; and as they must always be the nests in which disease, if not begotten, is nurtured, fed, and cherished, until it has acquired its fullest force and vigour, the proprietors should be compelled to keep them wind and water-tight, and to whitewash the walls twice a year; and the public should be taxed for the cleansing and purifying these houses. It is compulsory on us to feed and clothe the poor, for their sakes. It ought to be no less compulsory on us to keep them clean, and free from all the causes of disease, for our own sakes." (P. 66.)

Many of these suggestions might be acted upon with advantage, although some would be regarded as too arbitrary for this country. There are other regulations which should be enforced for the general benefit, such as the removal of dung and manure before the middle of the day; the conveyance of water by spouting from the roofs of houses; the removal of large signs hung across and obstructing the air in narrow streets; the removal of cattle markets out of the streets; and other obvious nuisances. The new corporations will possess ample powers to do this kind of good, and it will be the fault of medical practitioners if such representations are not made to the mayor, aldermen, and citizens, or councillors, or commonalty, or burgesses, as the case may be, as will bring about all these most desirable improvements. Whether it will be found practicable to establish public walks, play-grounds, baths, and places of healthy recreation and amusement in the open air, as contemplated by Mr. Buckingham in his Bill for these purposes, we can hardly take upon us to say; but we believe that the habits of the people, which present the principal obstacles to carrying such a bill into effect, might be so improved as to realize all the benevolent intentions of the honourable mover.

Certain peculiarities of style which pervade Dr. Kilgour's lectures might lead those looking cursorily over them to deem them trifling in their character, and intended as much to amuse as to instruct. We do him but justice, however, in stating that he appears to have exercised a sound understanding upon all the subjects upon which he has exercised his pen and his humour. To take examples from each of his chapters would be but again to carry the reader over ground already traversed; although throughout the journey he would find Dr. Kilgour a very lively companion. His defence of flannel, a subject already spoken of in this article, is admirable; and his dissertation on dress is a sort of skirmish with the follies of men, women, and philosophers, all of whom he chastises with little scruple. We turned to the chapter on Exercise, expecting to find good things there, and have not been disappointed.

Pointing out the improvement of the mental powers which is caused by exercise of the body, he truly enough limits this observation to moderate exercise. The student, we apprehend, will always experience, not only that without bodily exercise the mind becomes languid; but that if much active exercise be taken, or if he be very much in the open air, the aptitude for mental exercise is lost, dissipated in mere physical happiness. Those who wish to live a life of study must take exercise until refreshment is produced, but stop short of excitement. Men of science have nothing to do with athletic sports. No fox-hunter is addicted to long-continued thinking. In these matters, as in all other matters, moderation is wisdom. Vigour of body, and strength of nerve, are to be courted in the air, and in the sun, and in active exertion. Mental preeminence demands the shade, the silent study, a tranquil unexcited body, and an exercised mind. Seeking either too ardently, we depart the farther from the opposite advantages: the hue of health and strength of muscle may be purchased by mental stupidity; and the finest pleasures of the intellect bought at the price of a sickly body and a shattered nervous system. The truest benefactors of mankind, and those whose faculties have been exercised for the greatest number of years for the good of others, have been men whose occupations led them to diversified habits; and all who desire to be equally useful should remember their example.

After speaking of the attitudes of standing, standing on one foot, kneeling, sitting, and the recumbent posture, Dr. Kilgour adds, in his peculiar way:

"With the exception of the recumbent posture, and in it only lying on the back, all these are accompanied with some muscular exercise, but they are exercises which affect only one set of muscles, the extensors. They are not so beneficial to the body as where extensors and flexors are alternately called into play, and they are fatiguing or exhausting instead of strengthening. 'Don't loll in that manner, Miss," bawls the kind mistress from her easy chair, to the young girl who has bent her body forward or to a side, in order to give some ease, and bolt upright again sits poor Miss to her task; but a weary and a profitless task it is, for it is a weary and exhausted mind, in consequence of a weary and exhausted body, which is applied to it, and she girds her stays the tighter next day to support her. Lolling is a heinous offence in schools, and to keep the mind *intently occupied*, and to prevent somnolency, the pupil is seated on a form without a back or a front, on either of which a support might be sought! The pupil of the Peripatetic philosopher was more fortunate than the inmate of the modern school. He got knowledge with exercise, and without exhaustion and fatigue; much better was he walking than sitting upright on a 'school form.'" (P. 187.)

Dr. Kilgour commends walking as the most natural and beneficial kind of exercise. A good walker, he says, is always a healthy person. Leaping is not to be recommended, and running is too severe an exercise. And here Dr. K. runs off into his jests. 'Smart walking,' he says, 'is quite sufficient; unless a person hereafter expect to have to run for life or liberty; and, in that case, a well practised pair of legs is of service. To distance an enemy or a deer is no bad thing, when one cannot conveniently knock him down.' So also, in advising the physician to accommodate the exercise he prescribes to the habits of the patient, he cannot avoid sliding into his customary pleasantries. 'It is not a matter of indifference,' he observes, 'to the recovery of the broken down constitution of a debauchee peer, whether he be sent to the tread-mill or to his shooting box; nor is it a thing of light moment whether the short thick legs of an obese, dumpy cit, covered with the usual breeches and stockings, carry him to his garden to prune his own trees, and watch his lilies and roses; or that the same legs, harnessed in close leathers, be sent on a tramp of some miles after the dogs, to be landed in a swamp whence the owner of these legs will not be able to extricate them.' Again, he sagaciously vindicates dancing, in the face of the prejudices of Dr. Willich, who, in a book which was in our younger days a great authority in quiet families, declares dancing particularly injurious, nay dangerous to females, and, deprecating the cooling process of the fan, advises the whole company after dancing is over, and before they venture into the open air, to change their *linen,* and afterwards to wait a quarter or half an hour, before they return home, taking meanwhile tea. 'It would really be amusing," observes the relentless Dr. Kilgour, "to hear the cry of 'the Marchioness' clean linen,' instead of the Marchioness' carriage, and my 'Lord Charles' fresh shirt,' instead of his cab.' Fencing finds much favour with him. The pupil of the dancing-school he regards as ever the mere creature of art; but the fencer has nothing of this: "he is equally without the lout of the raw bumpkin, and the grimace of the man that spends half his days neither in the heavens nor on the earth, but between the two." We apprehend that some of our readers may be matter-of-fact enough to require to be told that the man thus marvellously defined is no other than the dancing-master. Even in a foot note, the good doctor's wit deserts him not; he relates the anecdote of the Cardinal Richelieu being surprised by a courtier whilst taking what are called standing-jumps about his study; and slyly adds, "Jumping, and especially jumping *round,* is a favourite exercise with ministers and courtiers." Once more referring to Dr. Willich, who, speaking of the good effects of declamation, intimates that the exercise of the voice may be particularly salutary to the female sex, who are more confined at home than men; Dr. Kilgour lauds the scolding of servants, and "that much calumniated piece of domestic duty—the reading a curtain lecture." The observations of our author on Swimming, Riding, and Sailing, are sensible, and strongly expressed, and he gives honour due to Friction, an agent too much overlooked. To young children, especially during the period of dentition, friction and the tepid bath are, he considers, the safe-guards of life. "The professed *Rubber,*" he adds, "often meets with the *nasus aduncus* of the school-learned physician, but it would be well if this last learned gentleman would turn his scholarship to reading the many histories of cures by means of friction; and that he would recollect what Fuller says, that, 'Exercise is to physic as a bandage is to surgery, an assistance or medium, without which many other administrations, though ever so noble, will not succeed. Your regular pill, powder, and draught gentleman has a great contempt for rubbing; the effect of his ignorance."

In many modern works have been set forth the errors of bodily constraint committed in female education. They cannot be mentioned too often. Dr. Kilgour, quoting Dr. Cheyne's admiration of the earnest desire of romping, jumping, wrestling, and running, planted by nature in young persons, whereby, he says, their joints are rendered pliable and strong, their blood sweet and proper for a full circulation—exclaims in language of the strongest, but well worthy of attention :—

"Meditate on this, ye mothers, whose poor girls can scarcely walk, much less run and romp; and who procure for them crooked backs and pale cheeks. Meditate on it, ye parents who send your daughters to fashionable boarding-schools, in order that, in acquiring art, they may lose nature; and ye who are looking out for wives, say, will you take this deceptive creature with her pale cheeks, and fœtid breath, and distorted body—the victim of her mother and fashion—or her who comes bounding down the hill-side to your arms, with her ringlets streaming in the wind, her face with the freshness and glow of health, her body in the luxuriance and freedom of unchecked and uncontrolled nature, and her kiss sweeter than

"Sabean odours from the spicey shore,
Of Araby the blest."

(P. 201.)

We have given the reader sufficient specimens of the style of Dr. Kilgour to enable him to imagine how many clever things are smartly said upon Digestion, Food, and Drink; and far be it from us, in this dull age, to quarrel with an author for his jokes.

It is, we think, incumbent on regular practitioners to begin to pay a more systematic attention to Hygiène than has yet been done in this country. In France, in Germany, and in America, it constitutes a separate chair in the schools; with us it hardly gains attention in the sick room. Yet the importance to each patient of the air he breathes, the food he swallows, the exercises he takes, cannot be over-rated. The irregular practitioners, very heedless of medicine, pay more attention to these things, and gain thereby oftentimes the credit which should accrue to the man of science, who, deeply impressed with a sense of the powers of physic, is oblivious of the non-naturals. If this forgetfulness has not heretofore been without disadvantage, it must now be more detrimental to the regular practitioner's just fame; for there will soon be few houses into which Smith, or Combe, or Dunglison, or Kilgour, or Hodgkin, or some other teacher of hygienic precepts has not found his way; and they will be found more difficult to dispose of than was in times not long past by Buchan, the terror of the by-gone race of apothecaries. Dr. Kilgour, who is, indeed, not very sparing of his sarcasm, but whose sarcasm is often very well directed, reproves us all in good set terms for our avowed neglect. Speaking particularly of the *young* practitioner, he says—

"Whilst he is pondering on the case, weighing accurately in his mind the action of each medicine he is exhibiting, and watching with intense anxiety for its expected sanitory effect, the patient will quit this world very likely with the medicine in his bowels, but along with it some solid and substantial article that would require the digestive powers of the healthiest stomach. A hard bed is a "hard thing to a healthy person, and more especially to a fat female dowdy who measures all others' comforts by her own; and where rest might be life, death is hastened by following the advice of this feeling-hearted soul, in moving the patient for the purpose of shaking up his bed. A free ventilation might soon put him on his legs, and it would for certain expel effluvia; but open windows let in the cool air, and cold air is better felt than contagious effluvia; so the windows being kept shut, and the bed-curtains drawn close, the patient has the happiness of dying in an atmosphere of his own creating, raised to a proper putrifying temperature by means of a blazing sea-coal fire. What can we think of the man who, in circumstances like these, calls for paper, pen, and ink, in order that he may scrawl a receipt, in indifferent Latin, for worse medicine, and knows not to order that which would relieve the patient without pain or expense." (*Introd.* p. 4.)

The young practitioner will see that Dr. Kilgour is not very complimentary: but his advice is, notwithstanding that, by no means unworthy of being kept in remembrance.

Enough, we think, has been already said by us, in these observations of the several works we have noticed, to impress every reader with the utility of making hygiène not only a private study, but a part at least of medical education. This enlargement of the academical curriculum would certainly sometimes enable medical men to make a more advantageous appearance than they now generally do when called upon for public testimony. Their discrepancies, and the loose manner in which they express themselves, and the apparent want of fixed and guiding physiological principles, cause them to be looked upon as little better than paid advocates of one side of a subject. Nor can we wonder at this. The judicial annals of this country would furnish specimens of medical evidence so unphilosophical, so extravagant, and so suspicious, as to shake the credibility of the whole profession.

Any reader versed in the history of this country for the last forty years, must be able to call to mind more than one occasion in which a knowledge of the laws of hygiène might have prevented great public loss; and such loss would have been much greater and more frequent, if the intelligence and research of the medical officers of the navy and army had not led to the discovery of many of those laws by observation. But without taking this wide view of the subject, certainly no more useful object of enquiry can be presented to the attention than that of the means of *preserving* good health; without which all the gifts of fortune are deprived of their value, and life itself is a burthen. There are melancholy cases of disease against which no prudence would have been effectual; but their number is insignificant compared with that of those which spring from ignorance and neglect. By removing this ignorance, the instances of neglect will be made more rare; and nothing is more certain than that by increasing the general health of mankind, the general amiability and virtue, and thus in every way, the general happiness of human beings is increased at the same time. Great maladies, like great misfortunes, are borne with patience: the mind raises itself to a level with its duty, and attains the virtue of resignation: but the little hourly grievances of the valetudinarian, like petty evils, fret the soul without rousing it to dignified resistance, and make men at once miserable and contemptible. Now, the *little* evils are precisely those which hygiène can obviate; and if, beyond this, it can lessen the chances of occurrence of some of the greater maladies, it is most worthy of the consideration of every rational being.

The wealthy and the middle classes of society already enjoy, to a great extent, the advantages arising from those comforts which it should be the object of enlightened hygiène to secure to all portions of the community. Calculations which may be depended upon, go far to establish that position of Dr. Southwood Smith, of which we have already spoken, that longevity and happiness generally go together. In France, according to M. Villermé, the difference of mortality is signally observable; the deaths in some wealthy departments being only 1 in 50, and in some of the poorer arrondissements of Paris, 1 in 24 and a fraction; and, in the richer arrondissements, 1 in 41 and a fraction. According to Dr. Emerson, the deaths among the white inhabitants of Philadelphia are 1 in 42-3; but among the blacks 1 in 21-7. The Life Assurance offices of London present facts equally striking: Mr. Morgan found that the deaths which had occurred during thirty years, among 83,000 persons insured, were only in the proportion of 2 to 3 of what had been anticipated. The average of the annual deaths in the Equitable Society for twenty years was 1 in 81-5. That of the University Club, for three years, 1 in 86. Dr. Dunglison quotes these instances, and looks forward with a hope, which we fervently trust will be realized, to results no less satisfactory throughout the United States; where, he says, oppression is impossible; where equal laws and an extent and capability of country prevent any from perishing of want; and where each may, with temperance and industry, enjoy a condition which, compared with the condition of the wretched lower classes of many portions of the old world, may be called affluent. These, indeed, are glorious prospects. Nor is there anything unreasonable in them. Unless the social compact can effect this, what better is the fate of the civilized man than that of the wild hunter. Hewers of wood and drawers of water there may always be; but there can be no real necessity for these hewers and drawers being the prey of poverty, disease, and vice, to the end of time. To believe the contrary is opposed to the belief of a good Providence superintending the affairs of man; and so gross a belief is seldom found the associate of knowledge drawn from a contemplation of the works and ways of that Providence which ordereth all things in heaven and earth. If there is one truth more conspicuously written for man's instruction than another in God's government of the affairs of men, it is that where civil liberty and virtue prevail, where the laws are just, and the people are enlightened and industrious, —there diseases are fewer, life is longer, and happiness greater.

If, then, the philanthropist turns to the actual condition of the lower classes in the luxurious nations of the old world, as in our own, which presents enormous extremes of fortune, how is he to diffuse among them that knowledge upon which so many blessings depend. Whoever has been engaged in such attempts knows that the great difficulty is to find persons possessed of the requisite knowledge, together with the power of communicating it to those comparatively ignorant, and whose education has little prepared them for its reception. It is a just observation of the Count de Tracy, one of the most distinguished of the living metaphysicians of France, a country which has produced so many, that the great art of teaching is, to commence exactly at the point which the student has already reached. The discovery of this point is not always very easy. Besides which, there is with respect to many whom it would be desirable to induce to be learners of what relates to health and disease, another great difficulty, namely, how to reach them at all. Numerous books, and some of them of great merit, have of late years been prepared for the poor; but the poor never see them. They are read and admired by all, except those for whom they are intended, and whom they would most instruct. To bring useful knowledge into poor men's houses is still the great difficulty.

If we could flatter ourselves that our recommendation would be effectual with the numerous physicians and surgeons who may peruse these pages, we would earnestly impress upon them the duty of assisting in this great, humane, and we may truly say *patriotic* labour, of instructing the poor how to be healthy; and thus to facilitate their being virtuous, prosperous, and contented. Medical men alone can know and fully estimate the irrefragable evidence which justifies these expressions. They alone possess the knowledge which would be, to the poor, health and life. This great service would not be less worthy of them than those more laborious services which they now willingly give in hospitals and dispensaries to many who ought not to require the aid of charity. The truest, noblest, and most effective charity is that which teaches the poor to depend on themselves, to avoid the causes of disease, and, by preserving their health under all ordinary circumstances, to secure the means of providing for such visitations as care cannot avert.

From the works already reviewed in this article, any medical man may, with little trouble, derive materials for lectures to the poorer classes of his townspeople, and his local experience will always furnish him with illustrations. The public will be found grateful for such exertions. In a work on which we have not made any comment, that of Dr. HODGKIN, there already exists a model of the kind of lectures best adapted to the uninstructed classes, or to those who have had a little instruction and wish for more. We have not space left to do justice to these admirable discourses. They are but four in number, but they are literally *full* of information. The first lecture comprehends the subjects of air, light, cleanliness, and clothing; the second relates to articles of food, solid and fluid; the third is on the subjects of muscular motion and the intellectual faculties; and the fourth is on successive generations, and the education of youth. The mere enumeration of these subjects is sufficient to shew how well the lectures are adapted to the working classes; but it is impossible for us to convey to the reader a just idea of the variety and quantity of matter condensed into them, or of the simplicity and earnest philanthropy with which it is communicated. Dr. Hodgkin, who is well known to the profession as a pathologist, is a member of the society of friends, and to us, we confess, it gives no displeasure to see in some parts of the lectures, as on the subject of war for instance, some of what, in the present sophisticated state of many who profess and call themselves Christians, we suppose we must call an amiable *enthusiasm.*

Justly does Dr. Hodgkin exhort his hearers, the mechanics of Spitalfields, to furnish *by their conduct* a plain and irresistible answer to those who call in question the propriety and advantages of the institution in which he delivers his lessons to them; and who conceive that to cultivate the native talent, and to increase the knowledge of working-people, "is to render them unfit for the discharge of their duties, and to make them dissolute private characters and disaffected subjects."

After enforcing the necessity of pure atmospheric air to man's continued existence, by a narration of the dreadful events which took place in a single night in the black-hole at Calcutta, he points out the precautions by which, even in the smallest and poorest houses, a supply of air may be obtained; and how a little ingenuity will remedy the faults of refractory windows, doors, and fireplaces. He warns them, by some striking anecdotes, of the effects of the fumes of charcoal, and those produced by the fermentation of beer and other liquors; and by what is called the choke-damp in coal mines; teaching them how its presence may be discovered, and how it may be drawn off. This leads to the consideration of the effects of accumulated filth in certain states of the atmosphere: he quotes Sir John Pringle's authority concerning the destructive consequences traced to want of attention to camp-privies; alludes to the improved health of sailors since the cleanliness of ships was more regarded; contrasts the present state of London with that of cities in which the plague yet prevails, as in former times it prevailed in London; and points out the fact that the worst fevers which yet pay us occasional visits, first appear in large cities, such as London, Edinburgh, Dublin, and Cork. These considerations lead him to the subject of Spitalfields. This constitutes one of the parts of London into which, it has often struck us, that if a gentleman or lady from Grosvenor Square were removed blindfolded, it would be difficult, when they were allowed to look about them, to persuade them that they were yet in London. Who, that has ever explored the terminations of Drury Lane, or cast a hurried glance up the courts opposite to the very walls of Gray's Inn, or, when going to dine north of Portman Square, has caught a glance of Calmel Buildings, can have done so without being sensible of the stinging satire all these dens of wretchedness, disease, and wickedness, convey upon our boasted civilization and refinement! In Spitalfields the contrast is less violent; but it is still a melancholy district, much resembling the worst streets of some of our worst constructed manufacturing towns; inferior, indeed, to the worst parts of Birmingham. Avarice, against which all appeals are vain, has so ordered the size and arrangement of many of the houses, that all which the poor occupiers can do to keep out disease must sometimes be baffled. The want of under-ground drains can, however, in some degree, be compensated for by a careful attention to the unobstructed state of the superficial gutters, and to the cleanliness of the highways. Filth is sometimes permitted to accumulate for two or three months. Large and deep holes are often left in the pavement, where pools of stagnant and offensive water are formed, "which being excluded from the influence of the sun and wind, are never dried up by the greatest heats of summer, but are constantly present to assist, by maceration, in the corruption and putrefaction of the various animal and vegetable offals which are thrown into them." In such streets, Dr. Hodgkin has found it difficult to allow a patient recovering from fever to go out into the air; and we are glad to learn, by a note appended to the lecture, that there has been some improvement since it was delivered. The neglect of the streets always leads to neglect of the interior of the houses: to keep them clean is, indeed, a hopeless task; and thus the seeds of disease are introduced to the dwellings. Dr. Hodgkin represents how easy it would be, by half a day's work, "to purify and enliven the walls and ceilings by the very healthful application of white-wash or lime;" by which, in addition to cleanliness, *light* would be introduced into the houses. The advantages of bathing are then alluded to, and Dr. Hodgkin suggests that the vast quantities of waste hot water poured out from the numerous steam-engines employed in the manufactories, might be made the means of placing a warm bath "within the reach of the poorest individual, who finds the means of procuring hurtful and debasing indulgences." The lecture is concluded by some excellent observations upon clothing; and the subject of light is again referred to, with reference to its influence on the health.

The second lecture contains a great variety of information concerning the different kinds of food and drink. The destructive effects of spirit-drinking are forcibly dwelt upon, without exaggeration, and the following remarks will interest the medical reader.

"The fatal influence of intemperance in drink, is occasionally seen a little beyond the middle period of life, at which time persons are not very unfrequently subject to what is called climacteric decline. Some are favoured to recover from its attack; but to the spirit-drinker it almost always proves fatal. Premature old age is another result of spirit-drinking. I have often noticed, with surprise, in the course of my practice, that when I had suspicion of the habits of a patient, and have enquired his age, that with all the marks of age and decrepitude upon him, he was some years my junior. The habit of spirit-drinking unfits its victims to bear the wounds, fractures, and accidents of various kinds to which all are liable; and the skill of the surgeon is often baffled, or foiled, by the ill condition of his patient, who, by a long course of spirit-drinking, has destroyed the powers of his constitution. It is also worthy of remark, that the spirit-drinker is peculiarly susceptible of disease of all kinds, and, consequently, is likely to fall the first victim to fevers, or other epidemic distempers. The ravages of the cholera have confirmed this by unnumbered proofs.

"The heart and blood-vessels do not escape the injurious effects of ardent spirits. The former is subjected to great varieties of excitement, and the palpitations so produced may lead the way to permanent disease. Ossification of the valves, and thickening of the lining membrane, are the probable results. The arteries, both large and small, are very liable to become ossified; and when this effect is produced, the individual is very liable to apoplexy and gangrene. In a former part of this lecture, I have hinted at the injurious effects which improper drinks may produce on the lungs. There is, perhaps, no error of this kind by which this effect is so strikingly produced as when ardent spirits are taken. Besides the obvious effect which they must have in promoting and aggravating inflammation of the lungs, whenever these parts suffer from irritation, at a time when the system is under the influence of spirits, there are two other modes in which mischief is produced, affecting these organs, which are less obvious. First, it has been ascertained by experiment, that a greater exercise of respiration is required when the system is excited by spirit: hence, DIVERS cannot remain so long under water after they have been taking spirits, as they can at other times. Runners, also, find their wind shortened after drinking spirits. Now those who take spirits in sufficient quantity to affect the system, and then, under the excitement which they have produced, apply themselves to some laborious or active exertion, must expose the lungs, or organs of respiration, to the chance of very serious injury. The other effect to which I allude, may seem at first to be at variance with what I have just related, as well as opposed to the vulgar or common opinion respecting the effect of spirits. It is generally supposed that they promote the warmth of the body; on which account they are frequently taken by persons who have no inclination to intemperance, when they are peculiarly exposed to cold. This is a very fallacious practice. A transient glow may indeed be produced by the quickened circulation which for a short time succeeds the swallowing of the dram; but this afterwards becomes proportionally more languid; in consequence of which the surface, and more especially the extremities, become pale and cold, whilst the internal parts are both stimulated by the spirit, and loaded with the blood which has left the surface of the body. The object of maintaining and equalizing the warmth of the body is completely lost; whilst the internal organs are exposed to the danger of inflammation. This effect of ardent spirits is seen carried to its greatest and most dangerous extent in Russia, and other countries where extreme cold prevails. The inhabitants of these countries are apt to give way to the temptation to take spirits to an amount which produces overpowering intoxication. If, in this state, they expose themselves to the cold air, or are driven out of dram-shops and turned into it, the combined influence of the benumbing cold, and the liquor they have taken, produces a profound degree of torpor. Breathing, which is closely and necessarily connected with the production of animal heat, is almost suspended, and the individual, unless rescued from his dangerous situation, is soon frozen to death.

"The deleterious effect of spirit on the skin, is seen in the production of what are usually called grog-blossoms. Spirits, likewise, promote attacks of erysipelas, which is often severe, and even fatal, in persons whose constitutions are shattered by the use of spirits.

"The worst effects of spirits, as connected with bodily health, are those which it produces upon the nervous system; by which, I mean the brain and nerves. The first effect of a large dose of spirits on this system, is almost immediate, and quite notorious, causing swimming of the head, confusion of ideas, and staggering gait. The late Dr. Spurzheim, who is almost universally known, in consequence of the long-continued and close attention which he paid to the brain, declared that he had found brains peculiarly hard in this country, which he attributed to the general abuse of spirits. A striking, and often immediate, effect of intoxication, upon the brain, is apoplexy. When this is not immediately fatal, palsy is almost sure to remain. Epilepsy is another very serious disease of the brain, which, when not produced, may be greatly aggravated, by the influence of spirits. In females, they greatly promote a tendency to hysterics. One of the most serious diseases of the brain, brought on by the use of spirits, is called delirium tremens. Persons, whose age might induce one to suppose that they were in the prime of life, are sometimes carried off in a few hours by this dreadful malady. Those are the most liable to die from this affection who have kept up an almost incessant state of excitement by means of ardent spirits. It is not necessary that the quantity taken should have been such as to produce an extreme degree of intoxication. The individual may even have been able, in some degree, to attend to the various concerns in which he might happen to be placed; when, after the sudden removal of the stimulus, or the abstraction of blood, or some powerful influence on the mind, or sometimes without any assignable cause, a state approaching to madness, and often marked with tremors, muttering, and prostration of strength, suddenly comes on, and if not pretty promptly relieved by well-directed medical aid, is very apt to prove speedily fatal." (P. 153.)

From the remaining portions of Dr. Hodgkin's little book we find we must refrain from making any extracts, although in every page we observe something useful and something interesting. We gave honour due to Dr. Combe for representing the hard fate of governesses. We have to offer our meed of praise to Dr. Hodgkin for setting forth the unpitied lot of milliners. Condemned, like glass-blowers, or those who work in metals, or toil in manufactories, to breathe a heated vitiated air, they are still more debarred from exercise, and they are even kept longer at work. Almost daily experience shews the effects of this system to medical men, in the ruined constitution of young females. Enquire into their habits, and it is found that they go to work as soon as they rise in the morning, perhaps at six o'clock; that their meals are hastily taken, that they seldom or never walk out, but sit at work till eight, often till ten o'clock, and sometimes, when fashion demands more exertion from its slaves, till after midnight. Young as many of them are, they soon feel the effects of this regimen; and the after consequences on their health are very serious. Their first physical misery is indigestion; then follow amenorrhœa, debility, perhaps phthisis: or, if they survive, diseases of the liver, severe pains, anasarca, and ulcers of the legs. When to this we add that they are, as Dr. Hodgkin observes, deprived of the comforts of home and of domestic habits, that their occupation gives rise to a love of dress, and that this increases the danger of the temptations to which they are exposed, we have a lamentable picture of what may be suffered by one class of people in order to minister to the extravagance of others.

In our notice of the five publications of which the titles are prefixed to the present article, it will be seen that our commendation is scarcely qualified by anything in the shape of censure. If we were particularly anxious to display our critical perspicacity, we might, perhaps, point out some pages in the works of Dr. Southwood Smith and Dr. Combe that might have been omitted without disadvantage to the reader; and, availing ourselves of Dr. Kilgour's fearless style, we might have been more edifying in correcting its redundancy, or even in reproving the too free range of his subjects. We might have rebuked Dr. Smith for assuring his reader that the second sound of the heart is still generally referred to the dilatation of the ventricles; and we might have detected occasional misquotations from Byron and Shakspeare in Dr. Dunglison, and occasional blunders in Dr. Hodgkin's illustrative anecdotes, as in the celebrated one relating to Pitt and Dundas, which he has grievously spoiled. But this would have been unworthy of us. The design of these authors is so excellent, their knowledge is so extensive, and their good sense so conspicuous, that we can only congratulate the public on the possession of such able teachers. Differing from one another in style, and each pursuing a different route towards the same object, they will help to supply the want of useful physiological knowledge throughout all the gradations of society. Dr. Southwood Smith's work will afford matter for reflection to many meditative and well-informed minds; and Dr. Combe's treatise especially recommends itself to those engaged in the education of young persons of the middle and higher classes. Dr. Kilgour will gain the ear of numerous readers to whom a graver book, or one less abounding in forcible and homely similitudes, might be addressed in vain; whilst the magistrate and legislator will, perhaps, prefer the sedater matter in Dr. Dunglison's instructive volume. In each of the publications, also, medical readers will find much that is worthy of their notice, and there are many portions of the work of Dr. Dunglison, and also of that of Dr. Kilgour, which are particularly addressed to them, and contain much that they ought to be thoroughly acquainted with.

As each successive chapter of all these works has been the subject of our temporary consideration, the reflection has continually recurred to us, that of all the evils therein depicted as incidental to man, affecting different parts of his system, or meeting him in different parts of the world, the greatest number fall with the most certainty and the greatest weight upon the poor. To us, therefore, of all the works of which we have spoken, that of Dr. Hodgkin is the most deeply interesting. It is addressed to those who err not from vanity or from fashion, but from the necessities of their position, to those whose unsophisticated minds thirst for knowledge, and whose understandings are quite equal to the task of applying it to their every day life. The highest praise conferred upon an ancient philosopher was, that he had brought down virtue from the clouds and made her known to mankind. The next praise is due to those who bring wisdom from the schools, and lead her into private habitations. Much of this praise is due to each of the respected authors whose labours have been the subject of our comments; and it is especially due to the last-named writer, who has carried the light of knowledge, by means of his lectures at the Mechanic's Institution, into a thousand humble families; into byways, and courts, and alleys, too, where comfort seldom dwells, and where the throne of infection is set up; and where poverty and ignorance have so benumbed their victims, that they even require to be taught how to avail themselves of the common gifts of light and air.

Nor are there wanting considerations of much importance which should recommend the study of hygiène to individuals placed in circumstances of greater comfort; considerations on which the moralist, if not the physician, might forcibly and properly dwell. Medical men, who see more of the interior of society, and the details, if we may so call them, of domestic life, than any other class of observers, well know to what an extent happiness is abridged by mere infirmities of temper, and how often the peevishness, despondency, irritability, and discontent, which torment the social circle in despite of many respectable and even amiable qualities, are in a great measure the direct results of an imperfect attention to hygienic rules; less the product, in other words, of a bad disposition, than of a confined atmosphere, and indolence bodily and mental. Still more serious are the effects of negligence of these particulars on the character of the intellect, especially in middle age. No one who values the healthy activity of his mind, or wishes to accomplish great or useful objects; no one who dreads the invasion of frigid torpor and inactivity, and reflects on the possibility of surviving his mental life, should deem attention to his bodily health an unworthy care. To prolong existence is a far less worthy object of concern than to maintain the efficiency of the mind and body *during* existence: and, with this application, we may admit the wisdom of an ancient saying, that a wise man should be "careful of his health and careless of his life."

---

## Figures and Tables

**Fig. XXIII. f1:**
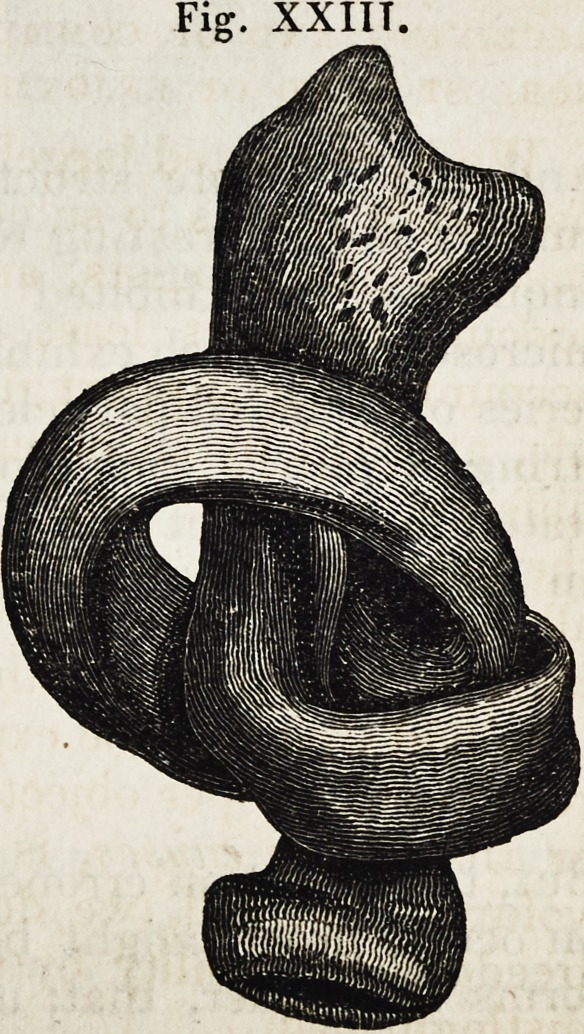


**Fig. XXIV. f2:**
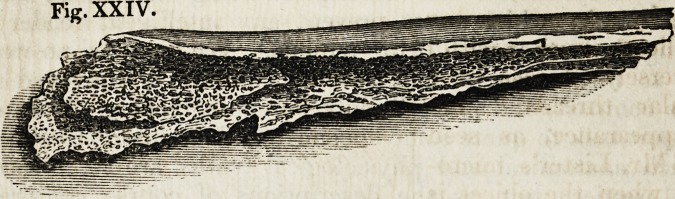


**Fig. XXVI. f3:**
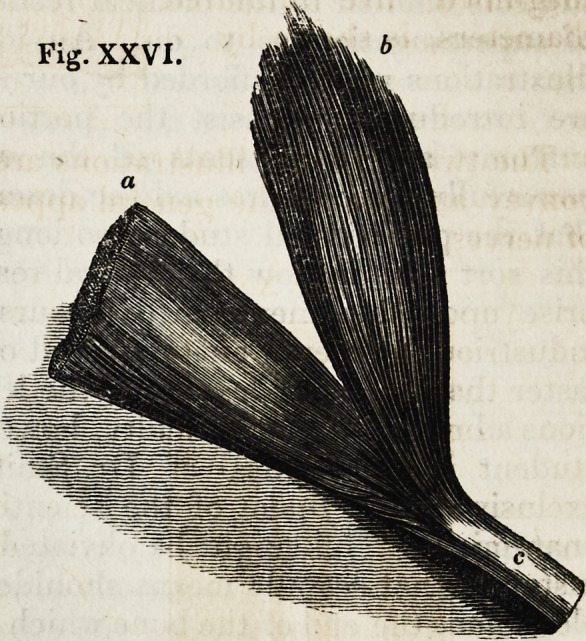


**Fig. XXVIII. f4:**
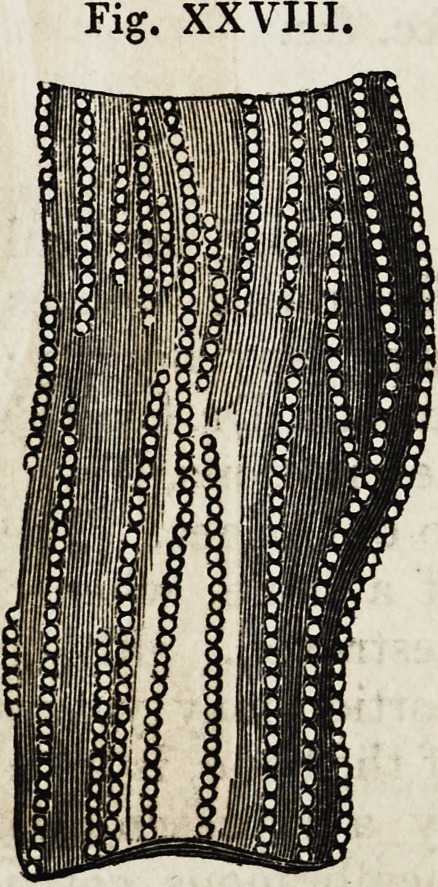


**Fig. XXIX. f5:**
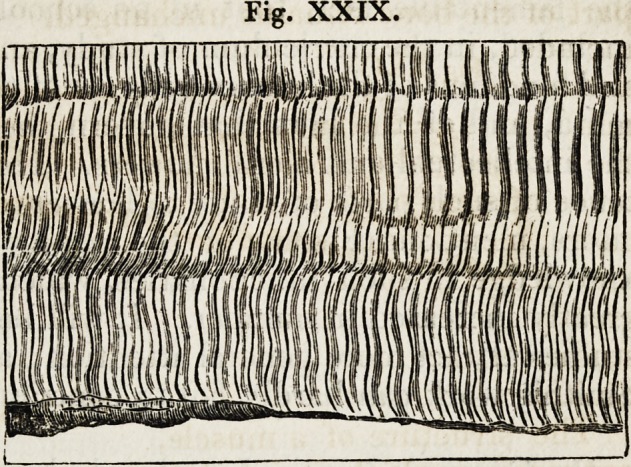


**Fig. XXX. f6:**
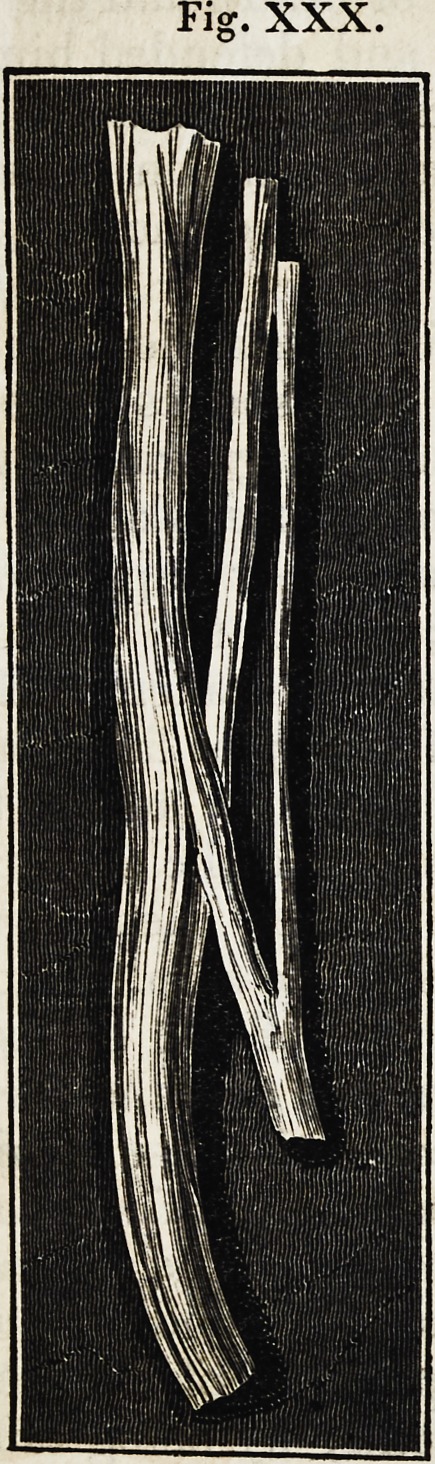


**Fig. XXXI. f7:**